# Parallel privacy preservation through partitioning (P4): a scalable data anonymization algorithm for health data

**DOI:** 10.1186/s12911-025-02959-z

**Published:** 2025-03-12

**Authors:** Mehmed Halilovic, Thierry Meurers, Karen Otte, Fabian Prasser

**Affiliations:** Berlin Institute of Health at Charité – Universitätsmedizin Berlin, Medical Informatics Group, Charitéplatz 1, 10117 Berlin, Germany

**Keywords:** Privacy, Data anonymization, Parallelization, Scalability, Utility

## Abstract

**Background:**

Sharing health data holds great potential for advancing medical research but also poses many challenges, including the need to protect people’s privacy. One approach to address this is data anonymization, which refers to the process of altering or transforming a dataset to preserve the privacy of the individuals contributing data. To this, privacy models have been designed to measure risks and optimization algorithms can be used to transform data to achieve a good balance between risks reduction and the preservation of the dataset’s utility. However, this process is computationally complex and challenging to apply to large datasets. Previously suggested parallel algorithms have been tailored to specific risk models, utility models and transformation methods.

**Methods:**

We present a novel parallel algorithm that supports a wide range of methods for measuring risks, optimizing utility and transforming data. The algorithm trades data utility for parallelization, by anonymizing partitions of the dataset in parallel. To ensure the correctness of the anonymization process, the algorithm carefully controls the process and if needed rearranges partitions and performs additional transformations.

**Results:**

We demonstrate the effectiveness of our method through an open-source implementation. Our experiments show that our approach can reduce execution times by up to one order of magnitude with minor impacts on output data utility in a wide range of scenarios.

**Conclusions:**

Our novel P4 algorithm for parallel and distributed data anonymization is, to the best of our knowledge, the first to systematically support a wide variety of privacy, transformation and utility models.

**Supplementary information:**

The online version contains supplementary material available at 10.1186/s12911-025-02959-z.

## Introduction

Sharing health data is crucial for advancing medical research, fostering innovation, and building trust in research findings [1, 2]. In particular, to fully leverage the potential of artificial intelligence and machine learning in medicine, we need comprehensive, large, and readily accessible datasets [3, 4]. In general, there is a willingness to share data, but this willingness often depends on the specific circumstances, data sharing scenarios and intended analyses [5, 6].

One particular challenge of sharing sensitive health data is the need to protect people’s privacy [7]. Many countries have laws to ensure privacy in shared data [8–10]. One approach to address this concern is through methods that make the data anonymous. This process, known as anonymization, has been the subject of extensive research, leading to the development of a wide range of methods [11].

The basic idea is to modify or transform data in such a way that privacy risks are reduced while the reduction of risks is balanced against a reduction of data utility. Several papers have shown that this is a complex task requiring care [12]. For instance, simply removing directly identifying attributes, such as names or social security numbers, will typically not be enough to prevent privacy breaches. More formal approaches, and additional safeguards [13], are required. Mathematical or statistical models can be used to quantify residual risks and the impact of anonymization on data utility and to trade both aspects off against each other [14]. A particular challenge for anonymization algorithms is to support a wide range of methods for transforming data and quantifying risks as well as utility, which is important as datasets, legal requirements and application scenarios can vary greatly.

### Objective and contributions

Algorithms to optimize the risk-utility trade-off can entail significant computational complexity [15]. This is particularly problematic when processing the very large datasets commonly found in medical contexts, such as those managed by health care providers, health insurance companies, and other related entities. Parallel and distributed algorithms are important mechanisms to support anonymization in such contexts and a range of approaches have been suggested (see Sect. “Comparison with related work”).

However, existing solutions often have the limitation that they have been specifically developed to implement certain transformation methods or privacy models. In this paper we present a simple, yet powerful, method for parallel data anonymization that supports a broad spectrum of methods. Our key contributions are as follows:

- We introduce a novel partitioning approach for distributed anonymization that achieves speedups through a utility-scalability trade-off, and which can be applied in both distributed systems, e.g., compute clusters, as well as to speed up anonymization on multi-core or multi-processor systems.
- Our method extends beyond existing approaches by incorporating additional steps to support a wide range of transformation methods as well as risk and utility models. First, records are sorted by their attribute values and partitioned for concurrent anonymization. In a subsequent merging phase, transformations are harmonized and re-applied where necessary, and compliance checks can trigger additional modifications to ensure the final dataset meets the specified privacy guarantees.
- We implemented the approach as open-source software in the multi-core setting, based on the health data anonymization tool ARX [16]. Our implementation supports 16 privacy models (see Sect. “Implementation“), as well as all utility (11) and transformation models (13) supported by ARX.
- We present the results of extensive experiments conducted to study the trade-offs taken. Across all our experiments a parallelization with 12 threads achieves a good balance between reductions in computation time (between 59.2% to 88.2%, corresponding to speedups between 2.45 and 8.46) and manageable reductions in output data utility (between 0.54% and 14.4%).

Our approach for distributed anonymization enables holders of large datasets to apply a variety of anonymization methods in a scalable manner.

## Methods

### Background

Anonymization algorithms aim to transform data in a way that lowers privacy risks for individuals below a certain threshold while optimizing the data’s utility. In addition to the removal of direct identifiers such as names, many approaches focus on risks resulting from indirect identifiers, also referred to as quasi-identifiers, such as age, sex, or zip code. The key idea is that these attributes, especially when combined, can form unique keys for individuals that can be correlated with other datasets. Moreover, some approaches focus on sensitive attributes, which contain information that does not necessarily increase the risk of re-identification but that individuals do not want to have disclosed about them.

**Fig. 1:**
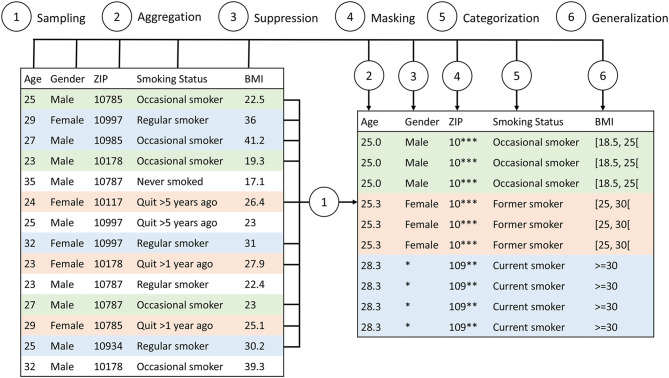
Example of common transformation methods. The input dataset on the left is transformed using a range of common methods to create an output dataset where each record is indistinguishable from at least three other records

As is illustrated in Fig. 1 possible transformations might include (1) random sampling of the records, (2) aggregating numerical values and replacing them by their mean, (3) suppressing values, records or complete variables, (4) masking, where parts of a string are obscured and (5) categorization as well as (6) generalization, which replace values with more general values. Categorization and generalization are usually performed based on user-defined rules for categorization or domain generalization. Transformations can be applied globally, which means that similar values will be transformed consistently across different records, or locally, which means that different transformations can be used in different parts of a dataset.

Transformations may reduce the fidelity of data or introduce uncertainty by introducing noise. The impact of these operations is usually captured by a utility model [14]. To quantify and formalize the protection provided by the anonymization process, privacy or risk models can be used. Privacy models address different threats, such as membership disclosure, attribute disclosure, and identity disclosure [14], and quantify risks for the individual records. Often, privacy models are based on different assumptions about the intent and background knowledge of adversaries [17]. Syntactic models estimate residual risks based on the structure of data, such as *k*-anonymity (see below) which uses the uniqueness of records as a proxy [18], while statistical models estimate relationships to the larger underlying population, such as the model by Dankar et al. [19], or the success probabilities of attacks. Semantic models, such as differential privacy [20], have more direct relationships to mathematical notions of privacy.

One example for a well-known privacy model is *k*-anonymity, which places restrictions on the distinguishability of data [18]. Here, the risk of identification or a record belonging to a specific individual is estimated through the distinguishability of the record from other records in the dataset. The parameter *k* acts as a threshold and specifies that each record must be indistinguishable from at least k-1 other records regarding all quasi-identifiers. Each group of indistinguishable records regarding the quasi-identifiers, forms a so-called equivalence class. Many privacy models use equivalence classes as a basis and formulate additional requirements for the records contained, e.g., regarding the distribution of sensitive values within each class.

In the context of this work and the approach developed, it is also important to differentiate privacy models in terms of their *monotonicity* [21, 22]. Two or more equivalence classes can be merged when the records contained in them have the same quasi-identifying variable values. A privacy model is monotonic, when merging two or more equivalence classes that fulfill the model always results in an equivalence class that fulfills the model as well. If a model is not monotonic, this is not guaranteed [22]. An example of a non-monotonic model is *t*-closeness [23].

### Basic approach

The basic idea of our approach, which we termed “Parallel Privacy Preservation through Partitioning” or *P4* for short, is to split the original dataset into partitions that then can be individually anonymized in parallel and merged into a common protected output dataset. However, this process needs to be controlled carefully, as depending on the types of anonymizations performed, additional steps are needed to ensure that the required privacy guarantees are provided.

**Fig. 2:**
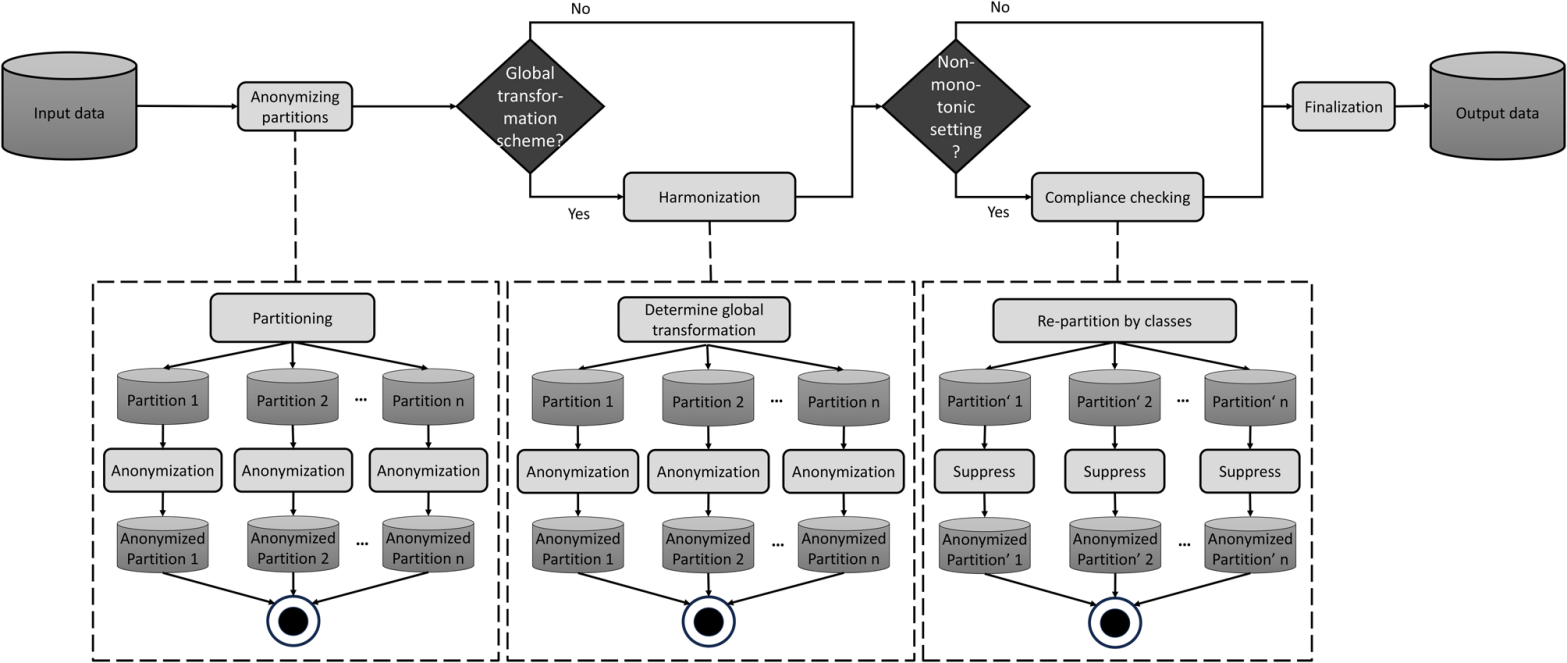
Overview of the basic steps of the P4 anonymization method

Our approach consists of four steps, which are illustrated in Fig. 2, and described in more detail below:

1. **Anonymizing partitions:** In this step, the dataset is partitioned into multiple disjoint datasets. It is a natural choice to choose the number of partitions to match the degree of parallelism to be used during processing. Multiple partitioning strategies can be implemented, e.g., range-based partitioning, clustering records by similarity or based on some form of ordering. Each partition is anonymized individually and in parallel using the same anonymization settings that are specified for the entire dataset, which include one or more privacy models, one or more transformation methods and a utility model.
2. **Harmonization:** If a global transformation method has been specified, the transformations applied to the individual partitions need to be harmonized. For example, if global attribute suppression or attribute generalization is being used, a common generalization scheme must be computed for all partitions and applied. Again, different strategies can be implemented, e.g., choosing the minimum, median or average generalization degree across all partitions. Each partition is then anonymized again, applying the harmonized transformation scheme.
3. **Compliance checking:** If a non-monotonic privacy model has been specified, equivalence classes that would fall together when merging the partitions (cf [22].) need to be checked for whether they are compliant with the requirements expressed by the privacy models. If they are not, they are removed from the output dataset.
4. **Finalization:** The anonymized partitions are merged and returned as the result. Optionally, this step can include performing a utility assessment.

We note that this approach implies an inherent trade-off between the degree of parallelism and the utility of output data. The reason is that anonymization algorithms are usually able to better optimize output data utility if they can access the complete dataset. Moreover, additional removal of information can take place as part of the compliance checking step. With our approach, as the individual partitions are anonymized independently, there hence is an expected reduction in utility.

### Implementation

We implemented the P4 algorithm in Java based on the open-source health data anonymization tool ARX [16]. ARX provides a programming library that supports a wide variety of privacy models, transformation methods as well as utility models with almost arbitrary combinations. Our implementation focuses on the multi-core scenario, as the algorithm can trivially be extended to run within a cluster of machines, which will only introduce communication overhead that increases execution times linearly to the dataset size.

The implementation currently supports the following privacy models: *k*-anonymity [18], distinct-ℓ-diversity and recursive-(*c*,ℓ)-diversity [24], entropy-ℓ-diversity with two estimators (Shannon and Grassberger) [25], *t*-closeness with equal-distance and ordered-distance [23], *δ*-disclosure privacy [26], basic and advanced *β*-likeness [27], average re-identification risk [28], sample uniqueness, profitability [29], *k*-map with and without estimators [30] which also equals, *δ*-presence (with *δ*_*min*_ = 0) [19]. It further supports all transformation methods provided by ARX and illustrated in the example in Fig. 1 as well as all utility models.

Note that the compliance checking step ensures that even for non-monotonic privacy models the resulting dataset fulfils the privacy requirements. Since all supported non-monotonic privacy models require that each equivalence class meets the privacy requirements, removing all (merged) equivalence classes that do not, ensures that the final dataset does.

As a partitioning strategy we implemented *lexicographical partitioning*, where records are sorted by the values of their quasi-identifiers in lexicographic order and then split into partitions. As a harmonization strategy, we implemented a method to calculate the *average generalization level* if this type of global transformation is being used. We found both methods to work very well in our experiments (see Sect. “Results”).

The implementation supports all utility models provided by ARX, but calculating a final overall utility estimate can be challenging. In our experiments we hence used a model capturing data fidelity by measuring the degree to which output data covers the domain of the original input variables (see Appendix D of [16]). The formula for deriving a global result is described in the Sect. “Calculating data fidelity for partitioned datasets” of the Supplementary File.

### Experimental design

To demonstrate the scalability and flexibility of our approach and to study the inherent parallelism-utility trade-off, we performed a series of experiments with different datasets, different privacy models and different transformation approaches. In each experiment, we executed our algorithm with increasing degrees of parallelism and measured the overall execution time, memory consumption as well as output data utility. In addition, we measured the time taken within the individual steps of the algorithm. In all experiments, we used global and local transformation methods and the utility model described above.

We used two real-world datasets, both of which have frequently been utilized for evaluating previous work on data anonymization: (1) United States (US) Census, an excerpt from the 1994 census database [31], and (2) Health Interviews, results from the Integrated Health Interview Series [32]. The size of the US Census dataset is 2.46 MB with 30,162 records and the size of the Health Interviews dataset is 93.06 MB with 1,193,504 records. The datasets contain nine variables each, including demographic variables and socio-economic parameters as well as temporal and geographic information. For more details about the datasets and how we configured the anonymization process, we refer to the Section “Datasets Configuration” of the Supplementary File. We further extrapolated the US Census dataset using the approach suggested by Zhang et al. [33] to derive datasets with up to 85 million records and a size of 6.5 GB. The algorithm is described in detail in Section “Data extrapolation algorithm” of the Supplementary File.

Our experiments were performed on a server machine with two AMD EPYC 7502 processors (each having 32 cores, running with 2.5 GHz) and 512 GB DDR4 memory, Rocky Linux 8.5 as an operating system (kernel 4.18.0–348.12.2.el8_5.x86_64) and the OpenJDK Java Virtual Machine (JVM) in version 17.0.1.

## Results

In this section we present the results for a selection of privacy models: *k*-anonymity, *t*-closeness and average (re-identification) risk. For an overview of all results, we refer the interested reader to Section “All results” of the Supplementary File. We chose those models, as they have different properties and the results are representative for all experiments. The selection includes monotonic (*k*-anonymity, average risk) as well as non-monotonic (*t*-closeness) models and models that measure privacy on record-level (*k*-anonymity and *t*-closeness) as well as on dataset-level (average risk).

### Execution times, memory consumption and data utility

**Fig. 3:**
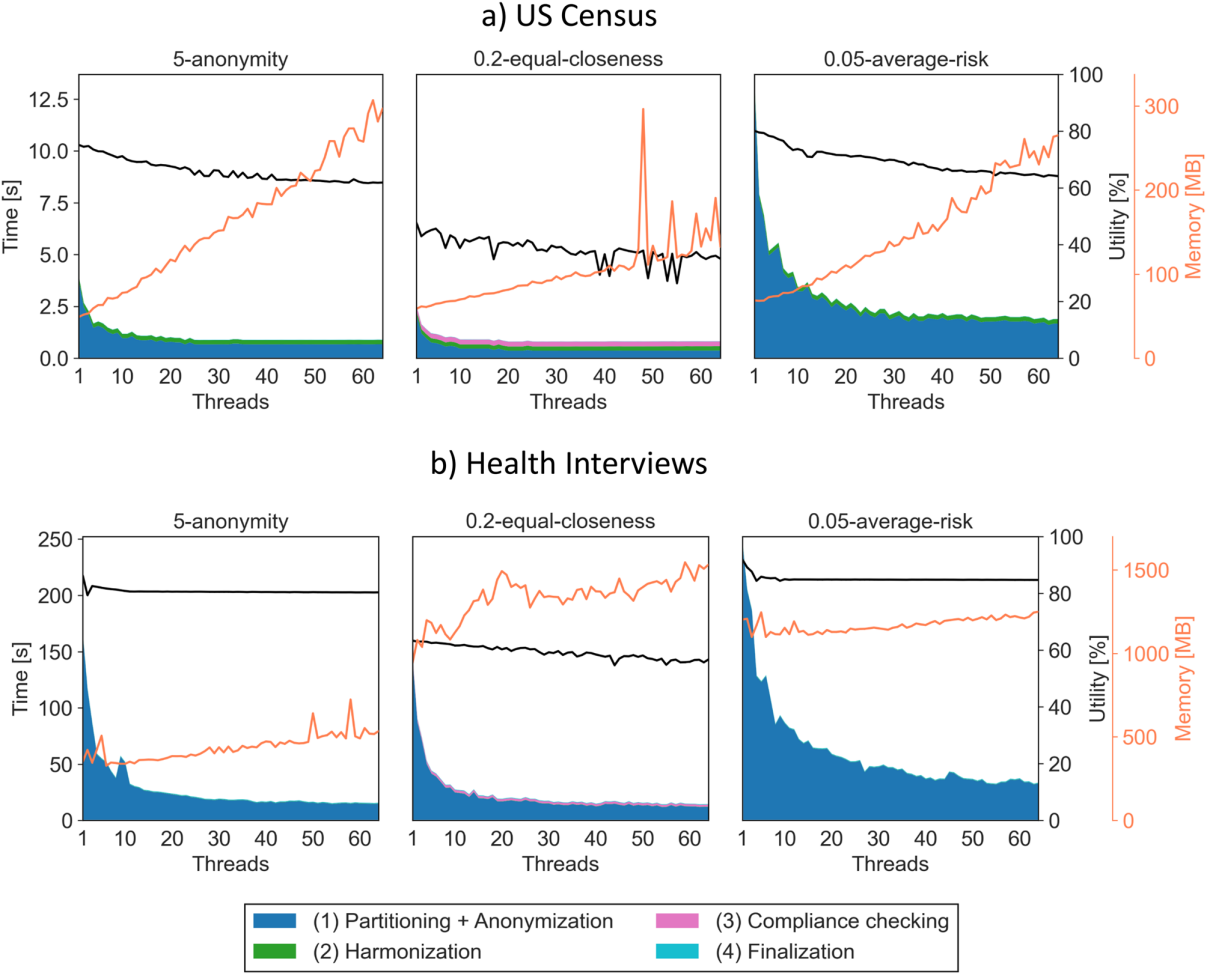
Results for the census dataset and Health Interviews dataset with the global transformation setting. The stack plot shows the time consumption of individual steps of the algorithm. The black line shows the development of output data utility and the orange line shows the memory consumption during execution

**Fig. 4:**
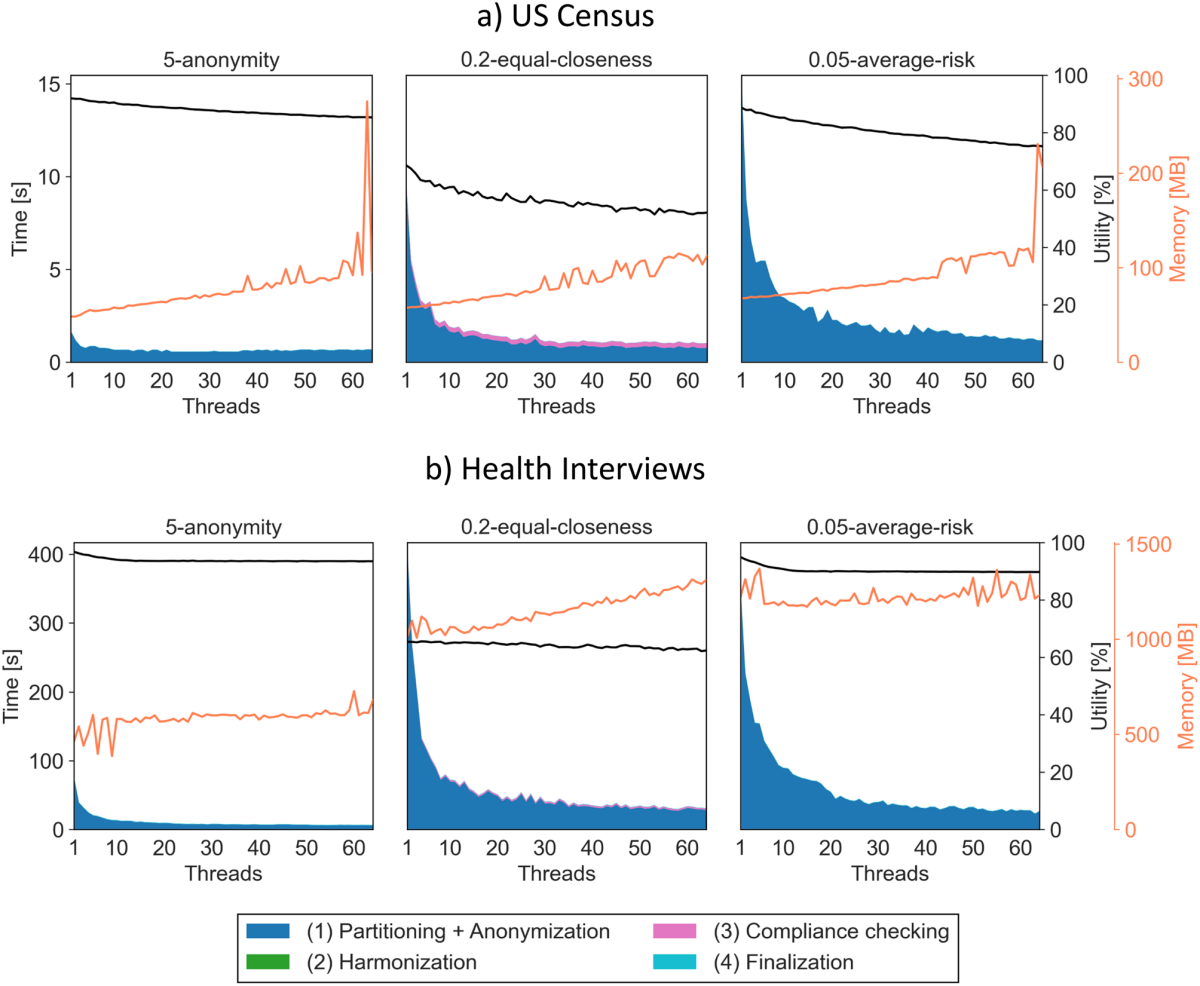
Results for the census dataset and Health Interviews dataset with the local transformation setting. The stack plot shows the time consumption of individual steps of the algorithm. The black line shows the development of output data utility and the orange line shows the memory consumption during execution

Figures 3 and 4 show results for (a) the US Census dataset and (b) the Health Interview dataset with the global and local transformation settings, respectively.

As can be seen from Fig. 3, which shows results for global transformations, the *anonymization* step is the driving factor for the overall execution time, which makes sense, as it captures the actual optimization processes being performed on the partitions. As the time needed to perform those optimizations depends on the size of the partitions, a significant speedup can be observed when increasing the degree of parallelism. For the small US Census datasets, the *harmonization* step makes up to 25% of the execution time, particularly with high degrees of parallelism, where the time needed for anonymizing the partitions is significantly reduced. For the larger Health Interviews dataset, the impact of the harmonization step is generally negligible. The same is true for the *compliance checking* step, which is only needed for the non-monotonic *t-*closeness model. The time needed to merge the partitions and calculate the overall utility is generally negligible.

For the small US Census dataset, with 2 threads execution times were improved by factors between 1.47 and 1.71, with 12 threads by between 2.75 and 3.68 and with 64 threads by between 3.04 and 7.13. At the same time, overall output data utility was reduced in relation to baseline utility achieved without parallelization by between 0.75% and 9.85% for 2 threads, 7.62% and 13.79% for 12 threads as well as 17.64% and 26.5% for 64 threads.

For the larger Health Interviews dataset, with 2 threads execution times were improved by factors between 1.21 and 1.6, with 12 threads by between 3.05 and 5.44 and with 64 threads by between 7.35 and 10.4. At the same time, output data utility was reduced by between 0.59% and 8% for 2 threads, by between 2.39% and 7.63% for 12 threads and by between 6.84% and 10.44% for 64 threads.

Figure 4 shows results for the local transformation experiments, where the *harmonization* step is not needed. Again, it can be seen that compliance checking can take a significant amount of the overall execution time when the degree of parallelism is high (up to 24%). Moreover, utility is generally higher in this scenario, as the local transformation model provides more flexibility in adopting to the data distribution (up to 21%). This additional flexibility usually also increases execution times (by up to a factor of 3.9).

For the small US Census dataset, with 2 threads execution times were improved by factors between 1.42 and 1.78, with 12 threads between 2.45 and 5.1 and with 64 threads between 2.4 and 12.8. At the same time, utility dropped by between 0.18% and 1.69% for 2 threads, by between 2.3% and 14.4% for 12 threads and by between 7.2% and 24% for 64 threads.

For the larger Health Interviews dataset, with 2 threads execution times were improved by factors between 1.53 and 1.91, with 12 threads by between 4.34 and 6.4 and with 64 threads by between 11.13 and 13.31. At the same time, utility dropped by between 0.04% and 0.85% for 2 threads, by between 0.54% and 4.99% for 12 threads and by between 3.41% and 5.5% for 64 threads.

Our results show a non-linear decrease in execution times. Generally, a degree of parallelism of around 12 seems to provide a good balance between speedups (between factors of 2.45 and 6.4) and manageable reductions in output data utility (between 0.54% and 14.4%). Moreover, the trade-off is better for scenarios in which local transformation methods are being used than in global transformation settings, where additional coordination is needed across the partitions (e.g., utility reduction of 0.54% for local transformation vs 2.39% for global transformation with 12 threads when implementing t-closeness for the Health Interviews dataset).

The optimization algorithms supported by ARX use a snapshotting mechanism, where interesting transformations of the dataset with certain properties are cached in memory to speed up analyzing future solution candidates [34]. This means that, as a rule of thumb, memory consumption increases with the execution time of the optimization process, which also increases for the “hardness” of the anonymization problem that is being solved. As can be seen in Figs. 3 and 4, we observed an expected linear increase, with a factor that is larger for the smaller dataset, which is harder to anonymize, and for *t*-closeness, which is also harder to satisfy than the other privacy models. The memory peaks measured in our experiments can be attributed to the JVM, where automated memory management can cause temporary memory spikes if enough memory is available. This behavior is expected, and we did not experience any memory-related issues or performance degradation, and the overall memory trends were well captured by our measurements. Overall, memory consumption was increased by factors of between 0.96 and 6.06 at 64 threads. Should memory consumption become a problem, ARX offers configuration parameters to control the amount of memory required.

### Scalability results

**Fig. 5:**
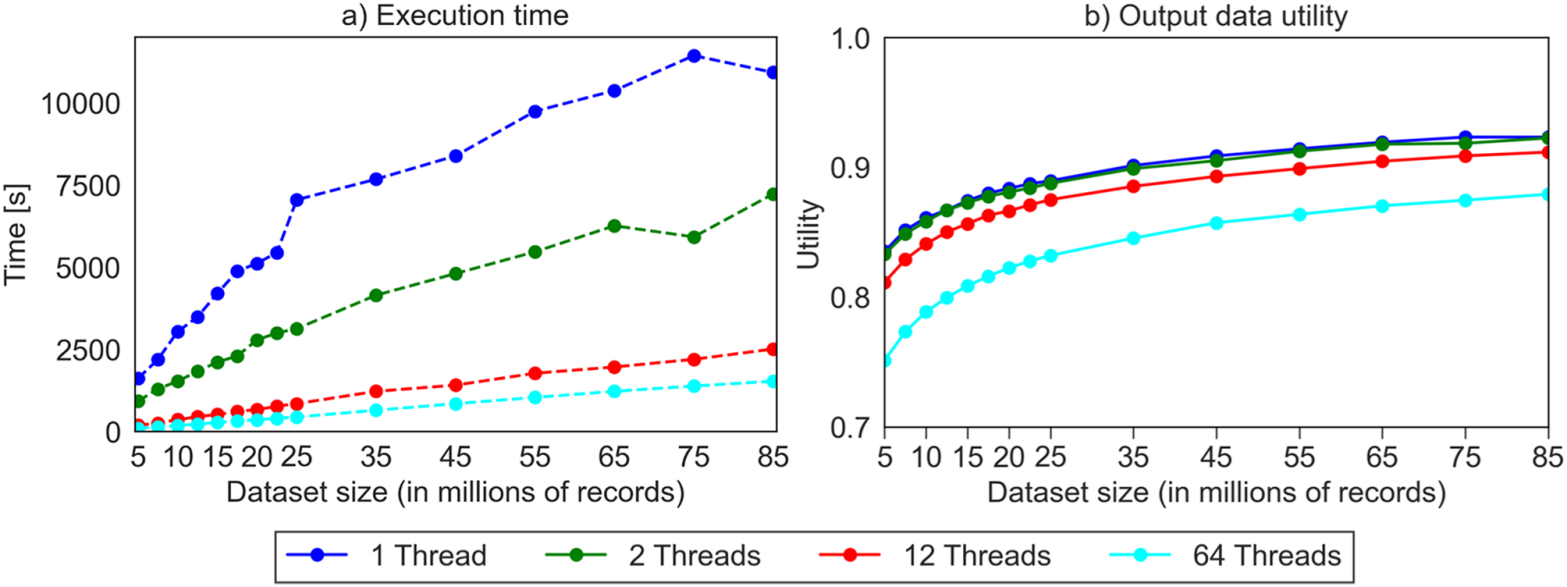
Results for extrapolated US Census datasets with increasing size

To study how our method behaves with increasing input data size, we utilized the extrapolated versions of the US Census dataset with numbers of records ranging from 5 million to 85 million. We used 50-anonymity as privacy model and a global transformation method. We choose this configuration to make our results comparable to one of the experiments conducted by Zhang et al [33], where a dataset with up to 25 million records was used. Figure 5 shows the results in terms of execution times and output data utility.

The results show that there is roughly a linear increase in execution time with increasing dataset size, while the factor is reduced when increasing the degree of parallelism. For the dataset with 10 million records, execution times were improved by factors of 1.99 (2 threads), 8.46 (12 threads) and 16.78 (64 threads), while utility dropped by between 0.28% (2 threads), 2.02% (12 threads) and 7.25% (64 threads). For the dataset with 85 million records, execution times were improved by factors of 1.51, 4.35 and 7.14, respectively, with a utility drop of 0.06%, 1.15% and 4.39%, respectively. Again, the results show that a degree of parallelism of 12 seems to provide a good balance between improved performance and reduction in utility.

## Discussion

In summary, this study introduces a novel parallel anonymization algorithm. Our experiments demonstrate an effective parallelism-utility trade-off, achieving significant reductions in execution times with acceptable reductions in data utility. With our implementation we support almost all (16) privacy models, as well as all utility and transformation models supported by ARX – in almost all arbitrary combinations. This enables users to configure the anonymization processes for a wide range of application scenarios.

### Comparison with related work

Previous studies have proposed distributed anonymization methods, which are not aimed at improving execution times, but instead support distributed data holders to jointly generate an anonymized dataset (see [35–37] as examples). In this section, we will focus on works that have proposed approaches comparable to ours and with the goal of improving scalability with respect to the number of records.

We note that scalability in relation to the number of attributes that need to be anonymized is an orthogonal problem that primarily affects the processing of individual partitions. In ARX, which is the basis of our implementation, this is already addressed by various heuristic algorithms, which efficiently handle high-dimensional data while maintaining strong anonymization performance. These algorithms have been extensively studied, and their effectiveness is detailed in prior work [38, 39].

Naturally, all related approaches work by splitting the dataset into partitions and working on each partition in parallel. For this, various partitioning methods have been proposed. One approach is to use random sampling to achieve partitions that mimic the distributions of the original dataset [33]. Another group of methods tries to create partitions, so that each partition contains records that are close to each other. An example for this is the quantile-based approach [40], which uses an ordering of an attribute to split the dataset into quantiles (or partitions), a method that is close to the approach used in our paper. The approach has also been extended to the multidimensional case [40], based on the splitting mechanism used in the Mondrian algorithm [41]. The Mondrian splitting mechanism has also been modified by Ashkouti et al. [42] to choose cut dimensions and cut points based on heuristics to create more balanced partitions. Later Ashkouti et al. [43] used a k-means based clustering to create partitions that preserve the requirements of the ℓ-diversity privacy model.

Most of the related algorithms are based on the Mondrian algorithm. De Capitani di Vimercati et al. [40] adapted the Mondrian algorithm to build a distributed anonymization method supporting *k*-anonymity and *ℓ*-diversity as privacy models. The method anonymizes the partitions and simply merges them, achieving reduced execution times by between 28% and 98%. Another algorithm based on Mondrian was suggested by Zhang et al. [44] and supports *k*-anonymity and differential privacy [20]. Using MapReduce, the approach iteratively splits the dataset until all partitions fit into the main memory of each computing node. Then the traditional Mondrian algorithm is used to anonymize the partitions and finally the results are merged. To support differential privacy, they modified their approach using the exponential mechanism and the Laplace mechanism [44]. Zakerzadeh et al. [45] presented an approach based on the Mondrian algorithm that also partitions the data, but uses a shared table of records available across partitions to achieve a better utility compared to other distributed approaches. Finally, Ashkouti et al. [42] also implemented a distributed algorithm for l-diversity based on the Mondrian algorithm, using the Apache Spark platform. The approach improves on the privacy utility tradeoff by creating balanced partitions using heuristics that take the distribution of all of the data related to an attribute into account. Zhang et al. [33] implemented a distributed anonymization algorithm using Top-Down Specialization (TDS) [46], which supports *k*-anonymity. Their method uses TDS on each partition to achieve *k*^*l*^ anonymity, where k^l^ > k. Then all intermediate results are merged and all steps are repeated until k-anonymity is satisfied. The authors showed that with tuned parameters, execution time can be reduced by almost 50% with a minimal decrease in utility [33].

The non-linear speedups observed in our experiments are consistent with previous results, for example by De Capitani di Vimercati et al. [40] and Zhang et al. [33].

Most previous work focusses on a small number of privacy models and only supports *k*-anonymity or ℓ-diversity [33, 40, 42, 43, 45] as well as differential privacy [44]. Notably, this means that the monotonicity property is irrelevant to those approaches and doesn’t need to be considered. Additionally, most of the previous approaches only support a small number of transformation methods, with all of them focusing on generalization and two approaches [40, 42] supporting additional transformation methods. All methods that are based on the Mondrian algorithm use local generalization, which means that same values for a given attribute can be generalized to different generalization levels, potentially resulting in a mix of concrete values and intervals. This can increase the difficulty of implementing downstream analysis tasks [47]. That’s why our aim was to propose a method that allows for a diverse set of configuration options for privacy and transformation models, including global and local transformations. Especially for health data this flexibility allows users to configure tailored anonymizations that likely will provide greater utility for planned tasks [17, 47, 48].

When sharing data, it is recommended to address privacy on multiple levels and to combine several safeguards as all methods come with residual risks (see [49] regarding health data anonymization). This is, for example, conceptualized in the Five Safes Framework [50], which describes five dimensions of privacy protection of which three are technical. A wide range of additional techniques can be used [51]. Techniques to address the Safe Data dimension include anonymization, as studied in this work, as well as pseudonymization [52], or synthetic data generation [53]. A Safe Setting for processing protected data can be constructed, for example through Federated Learning [54], which enables machine learning models to be trained across multiple institutions without transferring raw data, or Homomorphic Encryption [55], which allows computations to be performed on encrypted data. Safe Outputs, which ensure that results do not leak privacy, can be achieved by integrating Differential Privacy [20] into computations.

### Limitations and future work

One limitation of our work is that the experiments presented in this paper are based on a multi-threaded implementation of the approach. While translating the approach into a distributed setting within a cluster is straight-forward and the additional execution time needed for transferring data over the wire and performing disk-based partitioning can easily be estimated, this approach would still require the data to fit into the main memory of each computing node, as this is an underlying assumption of ARX. In future work we plan to develop a variant that also uses a disk-based anonymization procedure, which could result in quite different general properties.

Another limitation of our work is the utility model used in the experiments. We chose this model, as it is widely used and the results are easy to interpret. Moreover, with this approach it is straight-forward to derive an overall utility measure from the results for the individual partitions. While all utility models supported by ARX can still be used as an optimization function with our approach, deriving a combined utility measure for the merged dataset is non-trivial for many of them and would require additional work which we plan to take on in our future research.

Moreover, our implementation does currently not support all privacy models supported by ARX for multiple reasons. First, there are some privacy models, like hierarchical-distance *t*-closeness, that need statistical information about the overall dataset. While this can be integrated into our approach, it requires more work. Second, there are some privacy models, in particular those that extrapolate properties of the underlying population using statistical methods [19], where it is not trivial to see that they would work with a partitioning approach at all. Third, and notable, our distributed algorithm does not yet support the differential privacy implementation provided by ARX [56]. The reason is that it remains open whether and, if at all, how the global privacy budget needs to be split across the workers, which is another question that we plan to tackle in future research.

Finally, we plan to investigate approaches for further reducing the approach’s impact on output data utility. In this context, it could be interesting to study different partitioning and harmonization strategies. Moreover, integrating an approach similar to the work of Zakerzadeh et al. [45], which uses shared resources while anonymizing the partitions, could be investigated as well.

## Conclusion

In this paper, we presented the P4 algorithm, a simple yet powerful approach for parallel and distributed data anonymization. Notably, our approach is, to the best of our knowledge, the first to systematically support a wide variety of privacy, transformation and utility models. Especially in the medical context it can be helpful to tailor the transformations based on the available data and study goals. Our implementation comes with a parallelism-utility trade-off and a sub-linear decrease in execution times. In our experiments we observed that a parallelization degree of around 12 provided a reasonable balance. Our implementation is simple, uses the health data anonymization tool ARX and is available as open source software.

## Electronic supplementary material

Below is the link to the electronic supplementary material.

Supplementary Material 1

## Data Availability

All software developed as part of this project is available as open source at the P4 GitHub repository (https://github.com/BIH-MI/p4). Benchmarking results are also available in the repository (https://github.com/BIH-MI/p4/tree/main/evaluation/results). The datasets used for benchmarking are publicly available from the websites of the respective data holders (US Census: http://archive.ics.uci.edu/ml/datasets/adult, Health Interviews: https://nhis.ipums.org/nhis/), which are also listed in our repository.

## Abbreviations

JVM: Java Virtual Machine
P4: Parallel Privacy Preservation through Partitioning
TDS: Top-Down Specialization
US: United States
