# Supplementary material for: Parallel privacy preservation through partitioning (P4): a scalable data anonymization algorithm for health data
Source: BMC Med Inform Decis Mak. 2025 Mar 12;25:129. doi: 10.1186/s12911-025-02959-z (PMC11905666; doi:10.1186/s12911-025-02959-z)
Supplement: Supplementary file 1 — Supplementary Material 1 [file 12911_2025_2959_MOESM1_ESM.docx]

**Supplementary File**

Parallel Privacy Preservation through Partitioning (P4): A Scalable Data Anonymization Algorithm

# Calculating data fidelity for partitioned datasets

To measure the data utility of our output dataset we choose the utility model “Granularity”, which is described in detail in the Appendix D of [1]. To use the same notation, we also denote the number of records as *n* and the number of attributes as *m*:

$$Utility=1-{Loss}_{Data}$$

With:

$${Loss}_{Data}= \frac{1}{m}\sum_{1\leq x\leq m} \left( \frac{1}{n}\sum_{1\leq y\leq n} loss\left( x,y \right) \right)$$

Where $loss\left( x,y \right)\in[0,1]$ denotes the information loss per value, described as the fraction of the overall domain of the variable that the value covers, and is calculated depending on the type of the attribute $x$ and the transformation applied to the attribute. As the specific details of this information loss calculation don’t influence the subsequent calculation we refer the interested reader to Appendix D of [1] for more details.

Because of the commutative property of sums, we can write this as:

$${Loss}_{Data}=\sum_{1\leq y\leq n} \sum_{1\leq x\leq m} \frac{1}{m}*\frac{1}{n}loss(x,y)$$

Because of the distributive property, we can write this as:

$${Loss}_{Data}=\frac{1}{n}\sum_{1\leq y\leq n} \sum_{1\leq x\leq m} \frac{1}{m}loss(x,y)$$

Suppose we split the records in arbitrary sized nonempty disjoint partitions $P_{1}$ to $P_{k}$ and $\left| P_{i} \right|$ denotes the number of records in partition $i$ and $\left| P_{i} \right|>0$ . Then we get:

$${Loss}_{Data}=\frac{1}{n}\left[ \left( \sum_{y\in P_{1}} \sum_{1\leq x\leq m} \frac{1}{m}*loss\left( x,y \right) \right)+\ldots+\left( \sum_{y\in P_{k}} \sum_{1\leq x\leq m} \frac{1}{m}*loss\left( x,y \right) \right) \right]$$

Multiplication by identity for each partition with 1 = $\left| P_{i} \right|*\frac{1}{\left| P_{i} \right|}$:

$${Loss}_{Data}=\frac{1}{n}\left[ \left| P_{1} \right|*\frac{1}{\left| P_{1} \right|}\left( \sum_{y\in P_{1}} \sum_{1\leq x\leq m} \frac{1}{m}*loss\left( x,y \right) \right)+\ldots+\left| P_{k} \right|*\frac{1}{\left| P_{k} \right|}\left( \sum_{y\in P_{k}} \sum_{1\leq x\leq m} \frac{1}{m}*loss\left( x,y \right) \right) \right]$$

And this is simply:

$${Loss}_{Data}=\frac{1}{n}\left[ \left| P_{1} \right|*{Loss}_{P_{1}}+\ldots+\left| P_{k} \right|*{Loss}_{P_{k}} \right]$$

Which means that the utility measurements for individual partitions can be combined using a weighted sum to get a global utility estimate for the overall dataset.

# Data extrapolation algorithm

Our approach for extrapolating datasets is based on the method suggested by Zhang et al. [2]. With this approach, records are generated by randomly selecting a record from the original dataset and creating a variation of that record. For each attribute in the record, the method replaces the value with a certain probability with a value uniformly sampled from all possible values for that attribute. In the original method each record was varied a specific number of times and for each varied record each attribute value was replaced with probability ρ. As we want to generate datasets with predefined sizes, we instead generate N records by uniformly sampling a record each time to be the base for the new varied record. As suggested by Zhang et al. [2], we used a probability of ρ=0.66 to decide whether an attribute value needs to be replaced with a random value from all possible values for that attribute.

**Input**

- R: Reference dataset
- N: Number of records to be generated
- ρ: Probability that a value gets replaced

**for** (i = 0; i < N; i++) {

record 🡨 sample random record from R

**for** (attribute ∈ record){

**if** (coinflip(ρ)) {

record[attribute] 🡨 Uniformly sampling across
 all possible values for
 the attribute

}

}

**output**(record)

}

All results

In this section, we present the results for all experiments performed. As can be seen in Figure S1 to Figure S16 the trends observed in regards to execution times, memory consumption or output data utility, are comparable to those presented in the main manuscript.

**
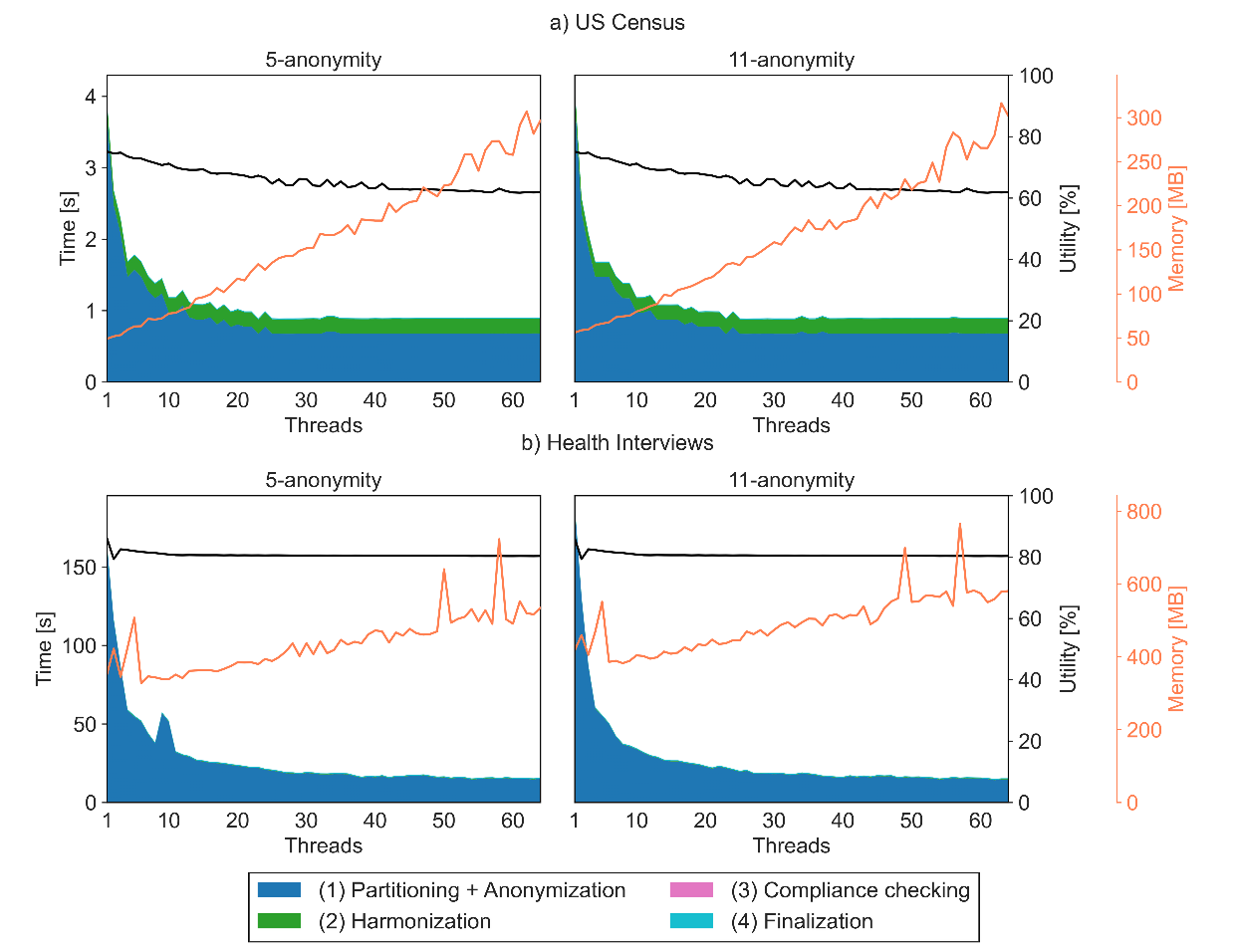

Figure S1:** Results for *k*-anonymity with the global transformation setting.

**
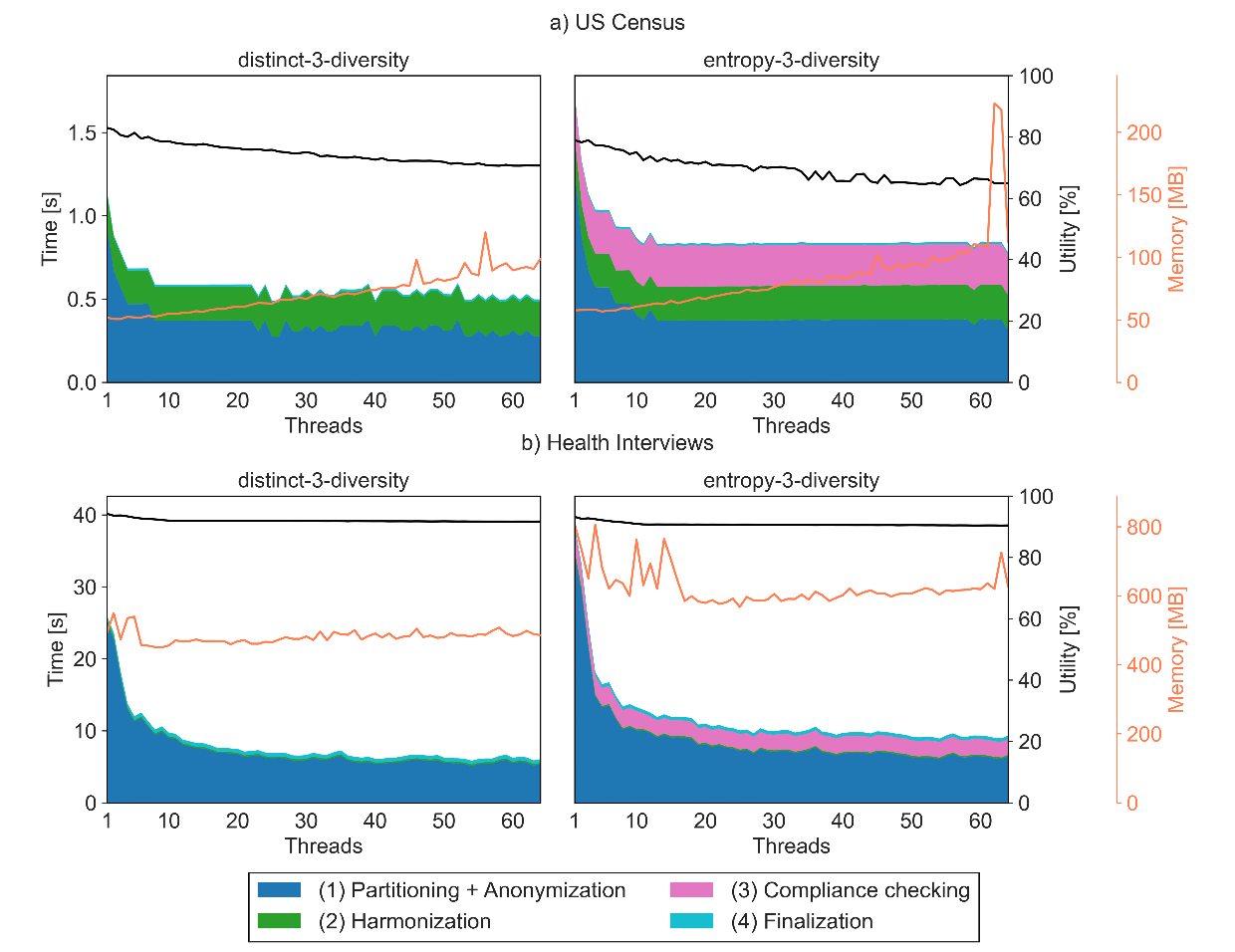

Figure S2:** Results for ℓ-diversity runs with the global transformation setting.

**
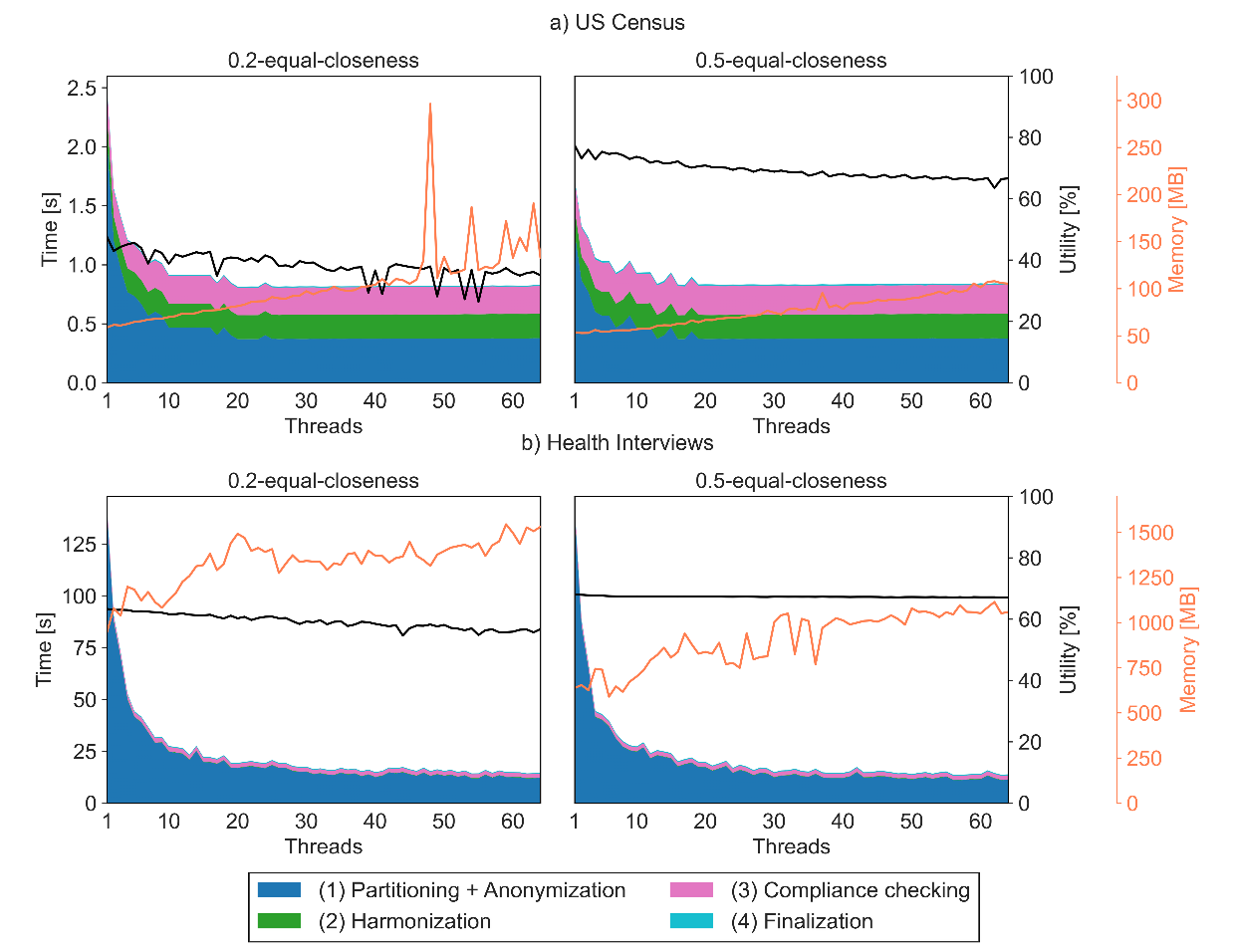

Figure S3:** Results for *t*-closeness runs with the global transformation setting.
**
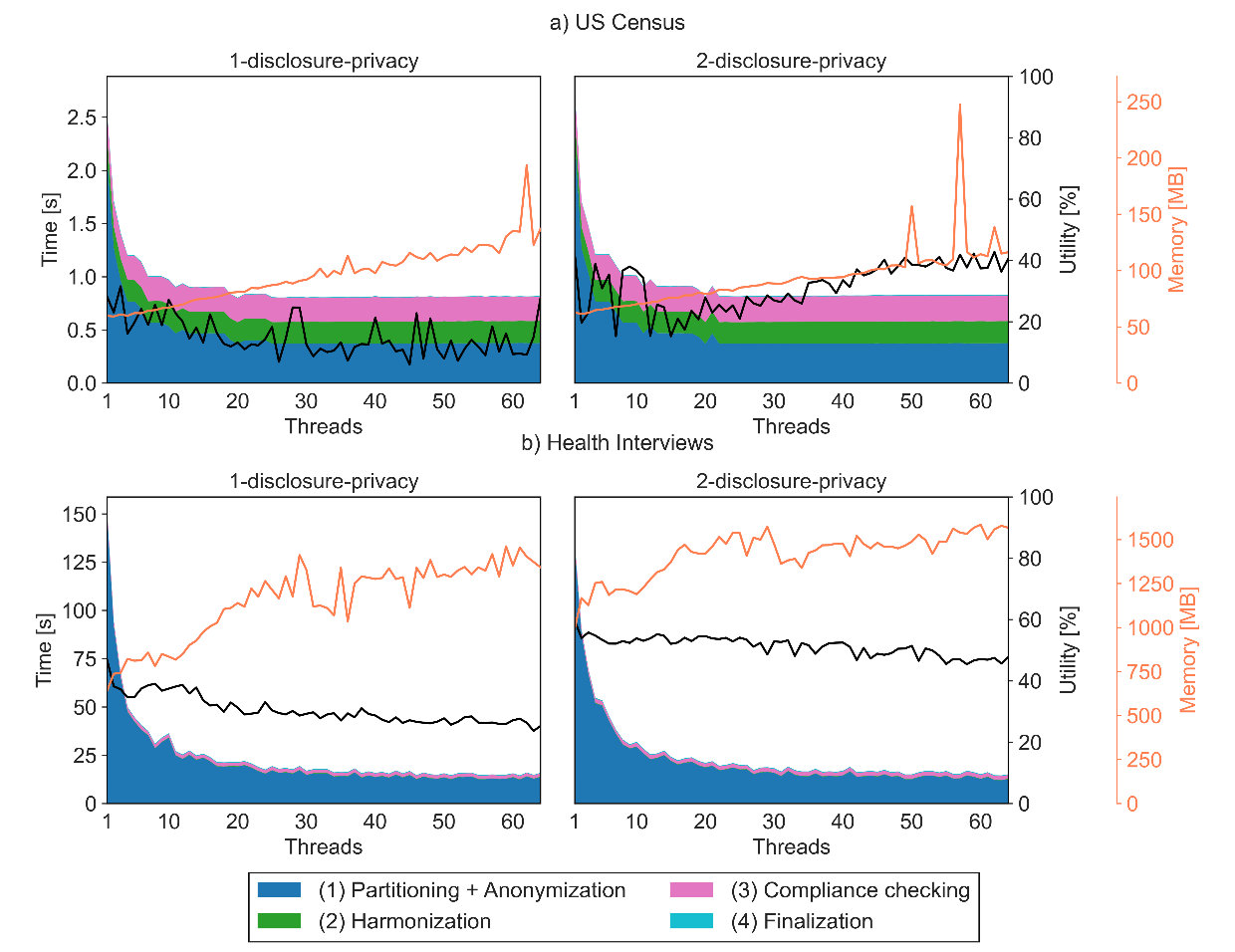

Figure S4:** Results for *δ*-disclosure privacy runs with the global transformation setting.

**
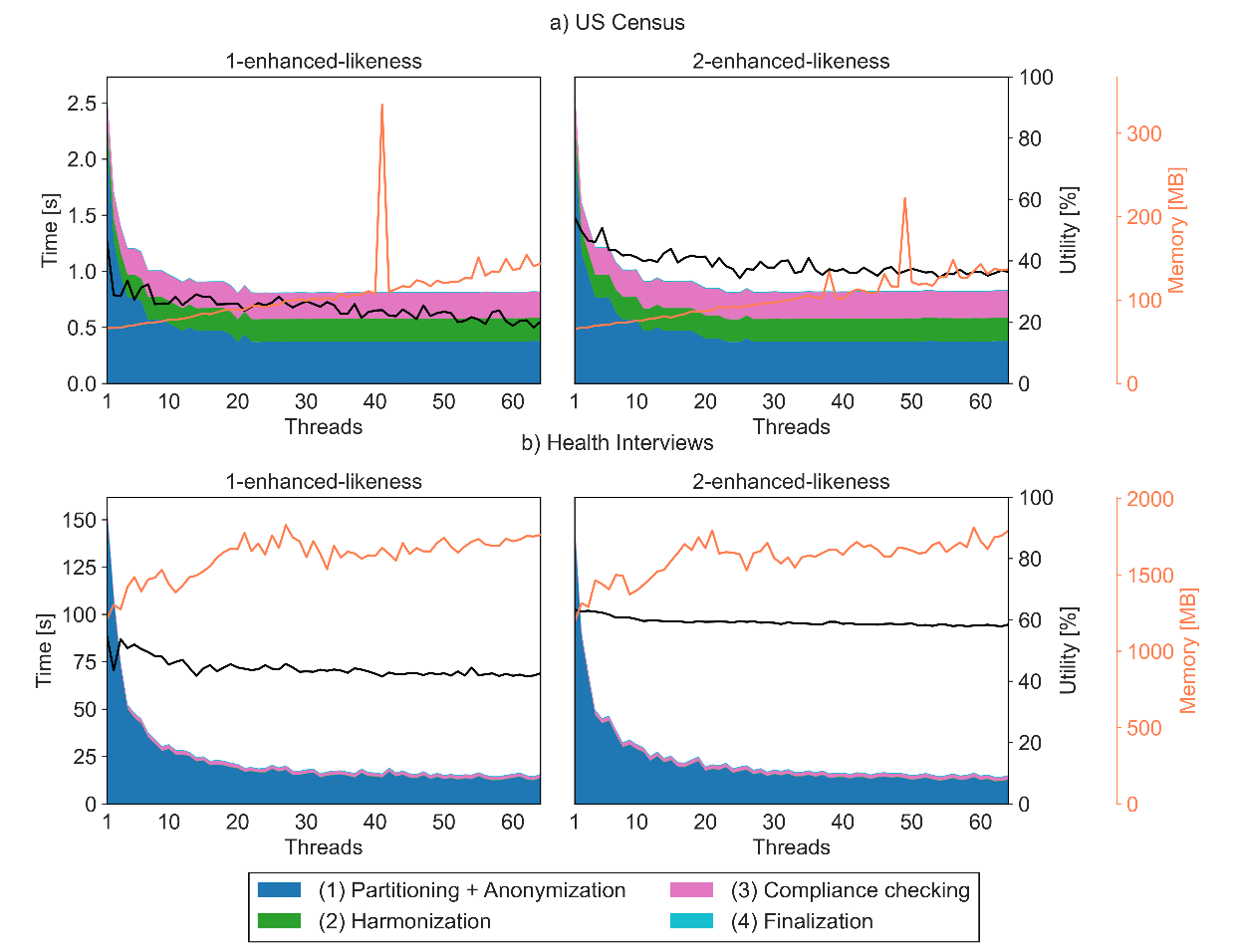

Figure S5:** Results for ⁠*β*-likeness privacy runs with the global transformation setting.

**
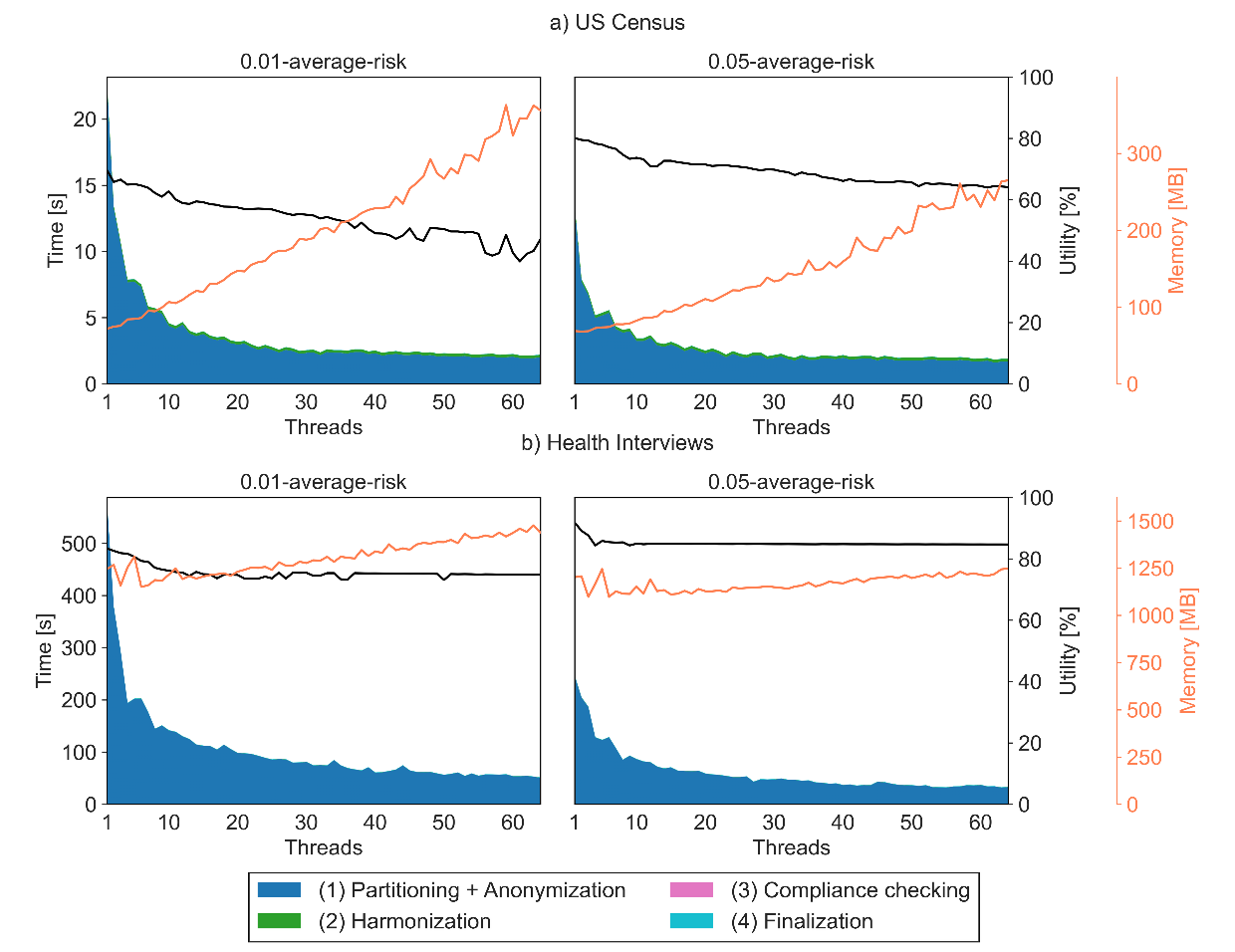

Figure S6:** Results for average re-identification risk with the global setting.

**
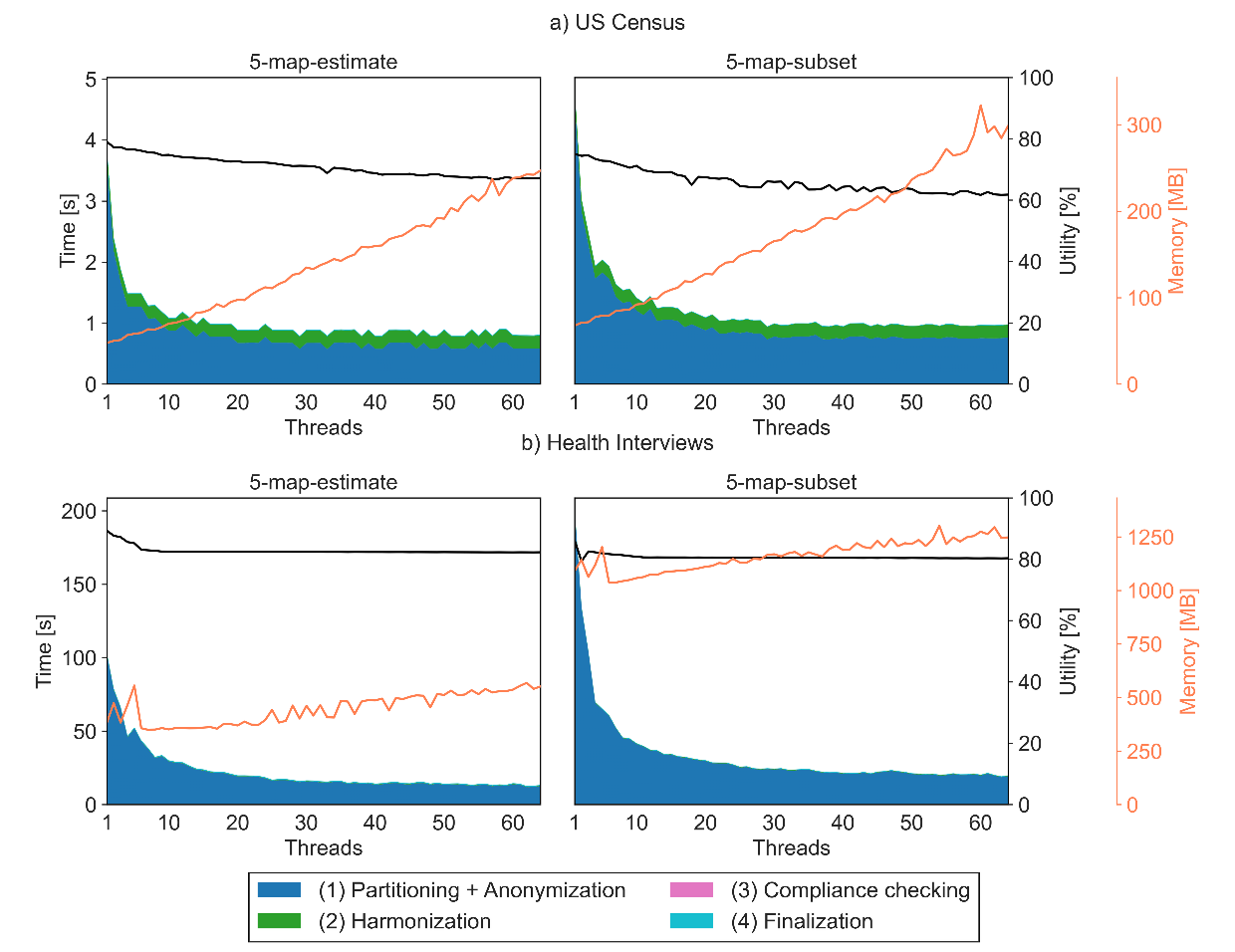

Figure S7:** Results for *k*-map with the global transformation setting.

**
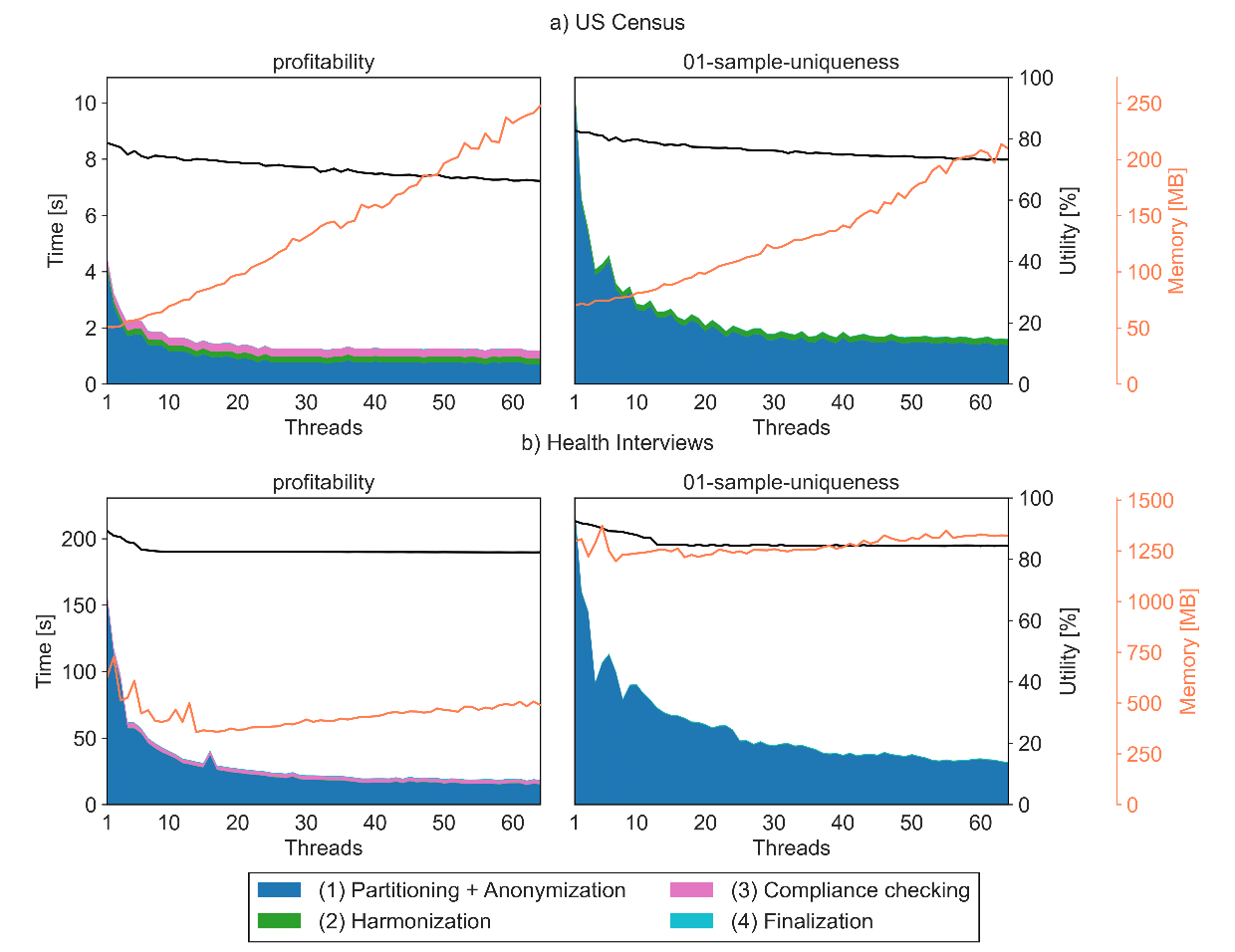

Figure S8:** Results for profitability and sample uniqueness runs with the global setting.

**
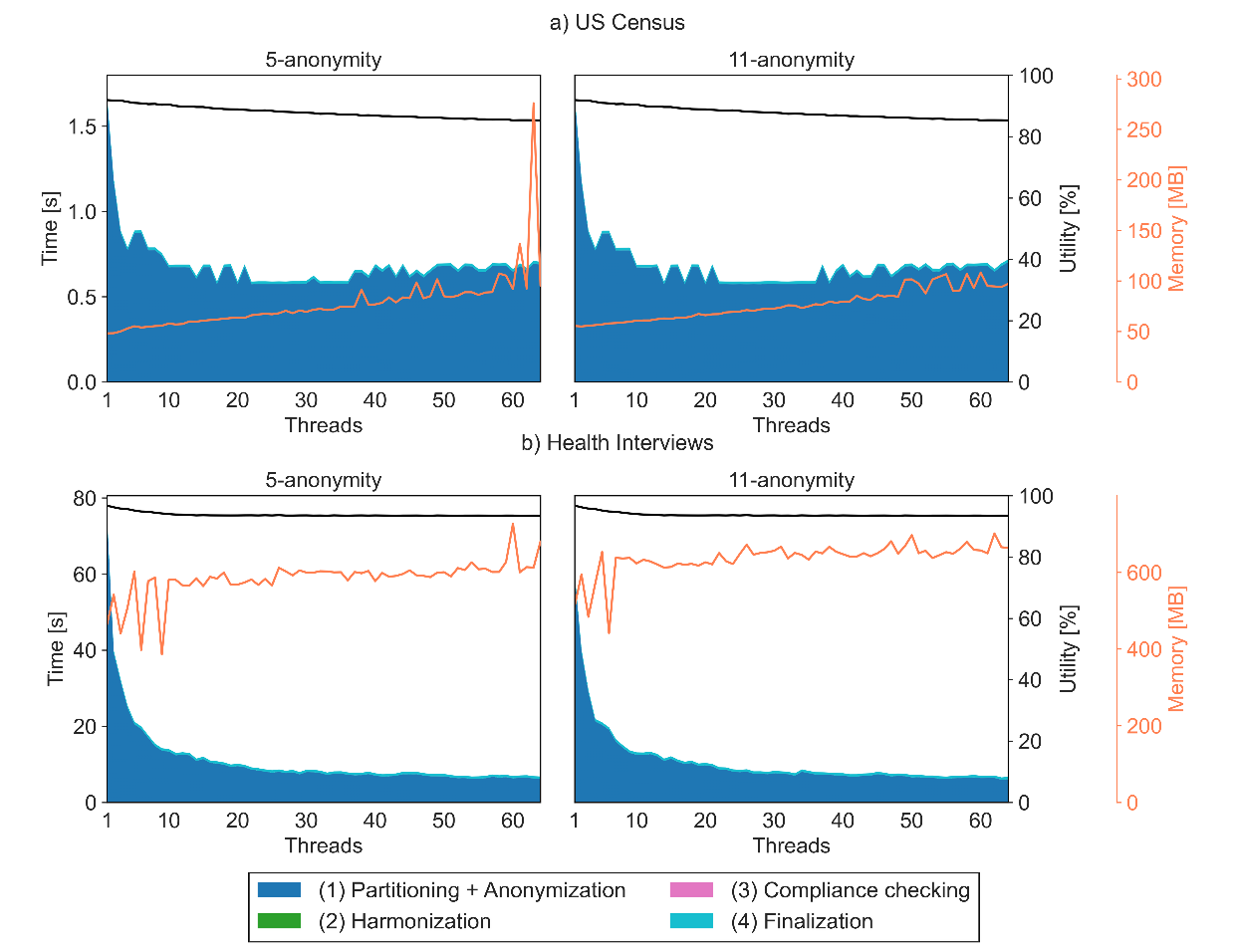

Figure S9:** Results for *k*-anonymity runs with the local transformation setting.**
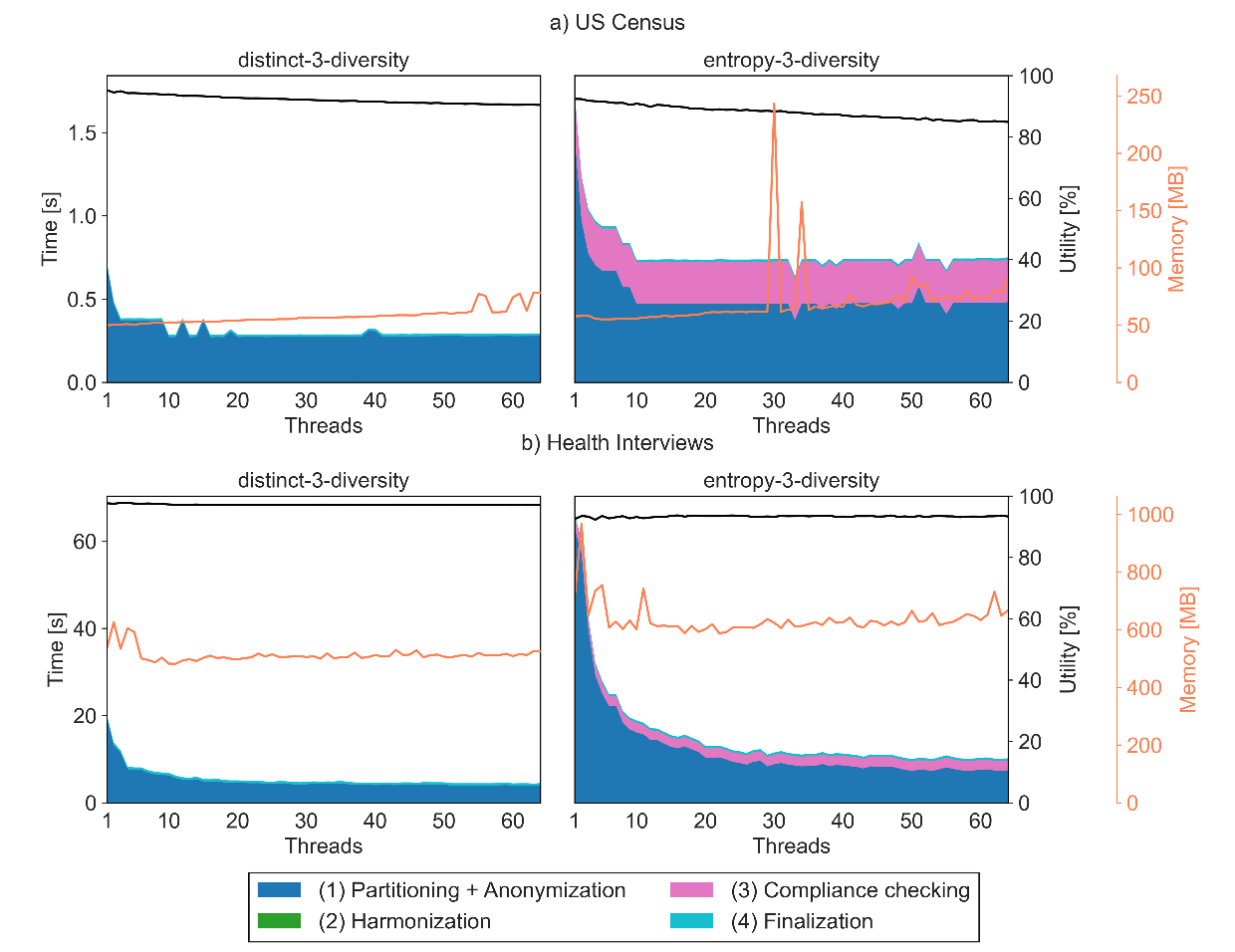

Figure S10:** Results for ℓ-diversity runs with the local transformation setting.

**
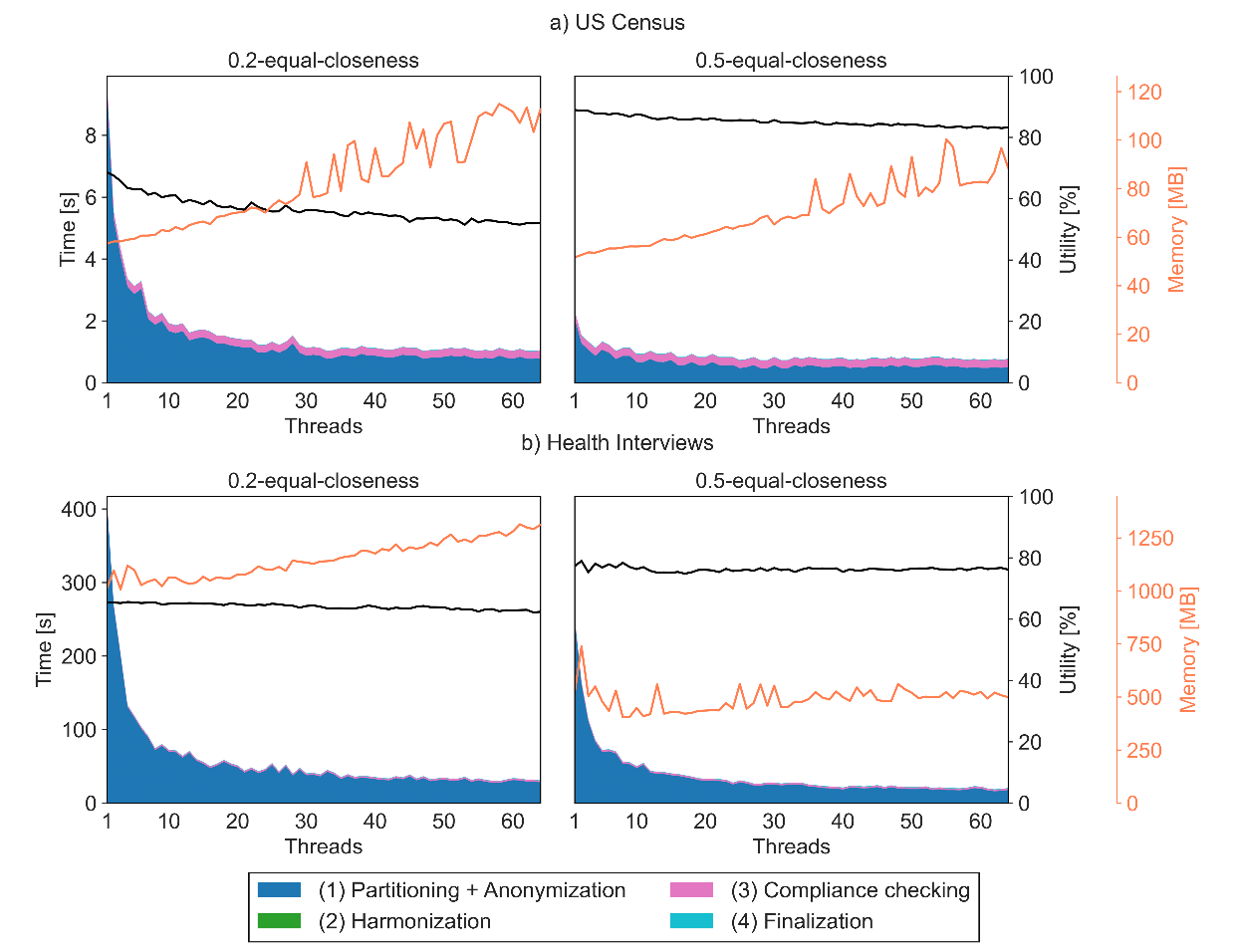

Figure S11:** Results for *t*-closeness runs with the local transformation setting.

**
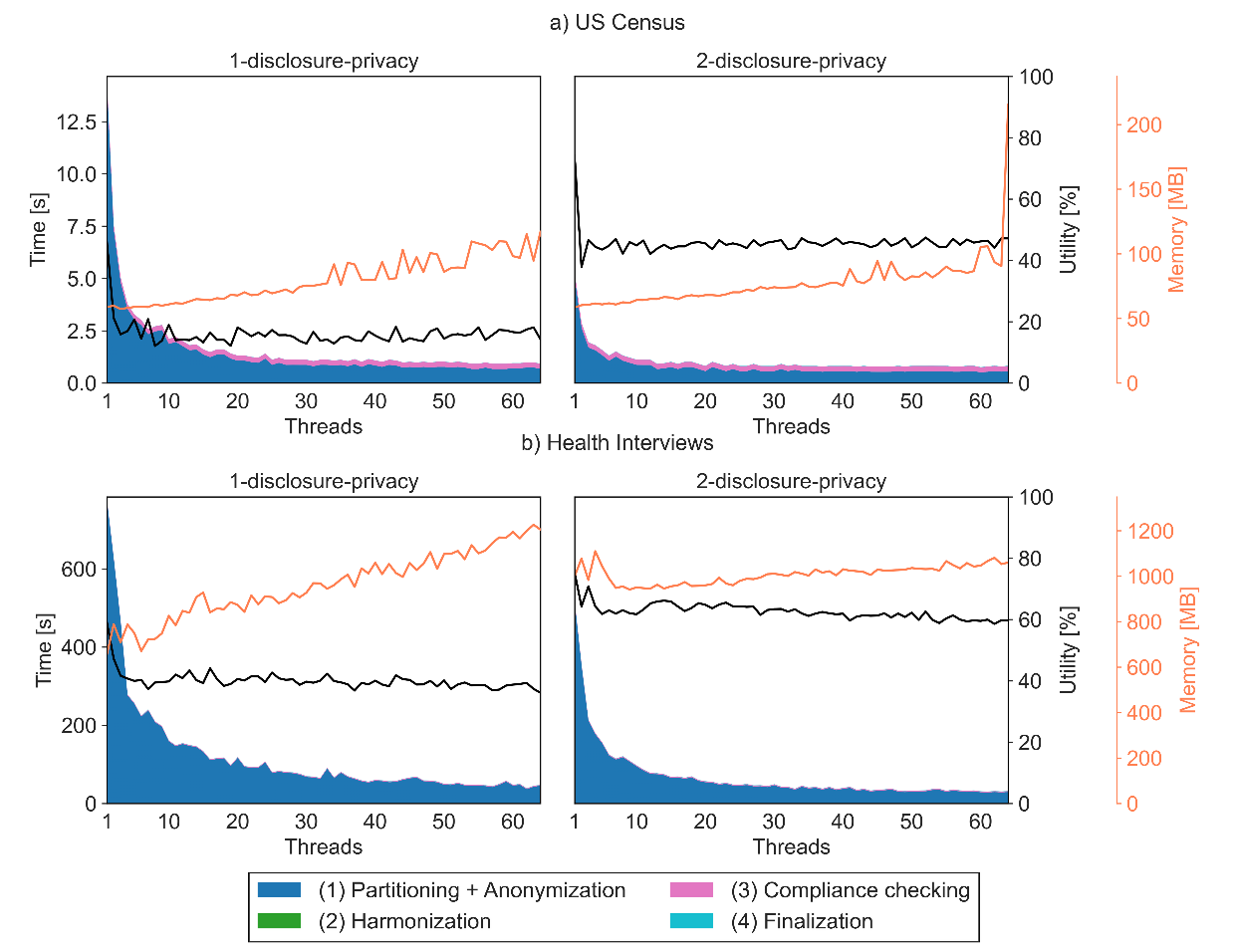

Figure S12:** Results for *δ*-disclosure privacy runs with the local transformation setting.

**
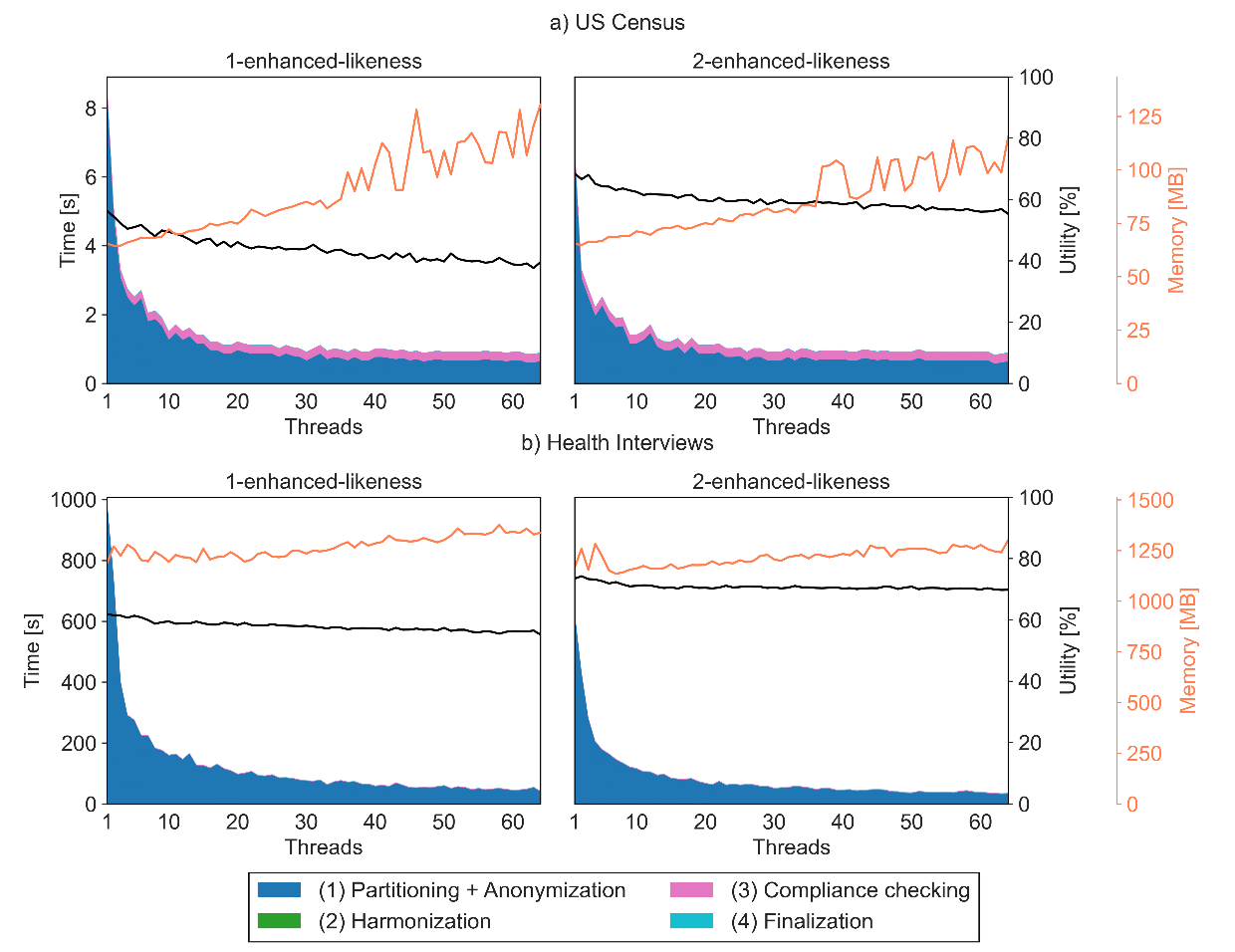

Figure S13:** Results for ⁠*β*-likeness privacy runs with the local transformation setting.

**
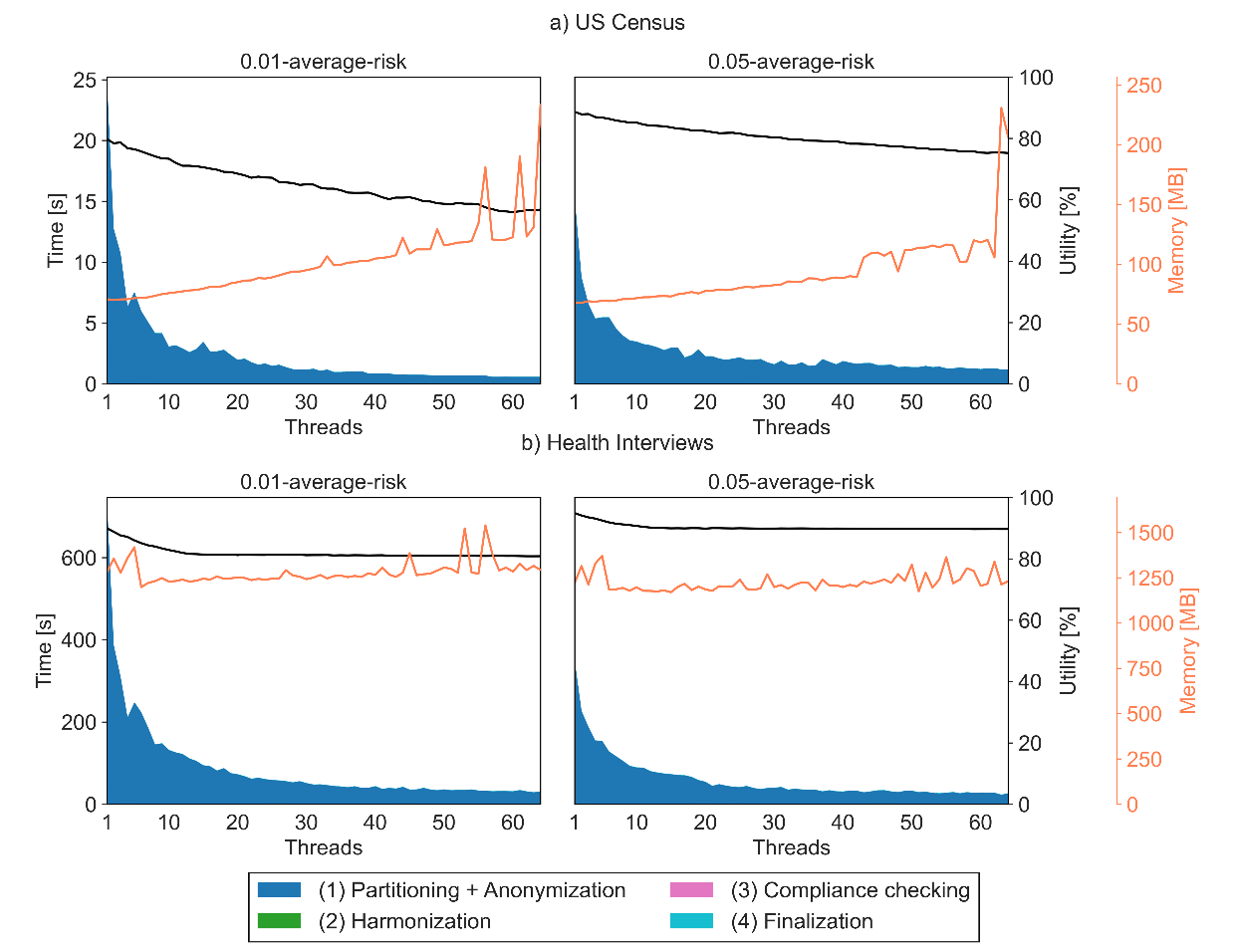

Figure S14:** Results for average re-identification risk runs with the local setting.

**
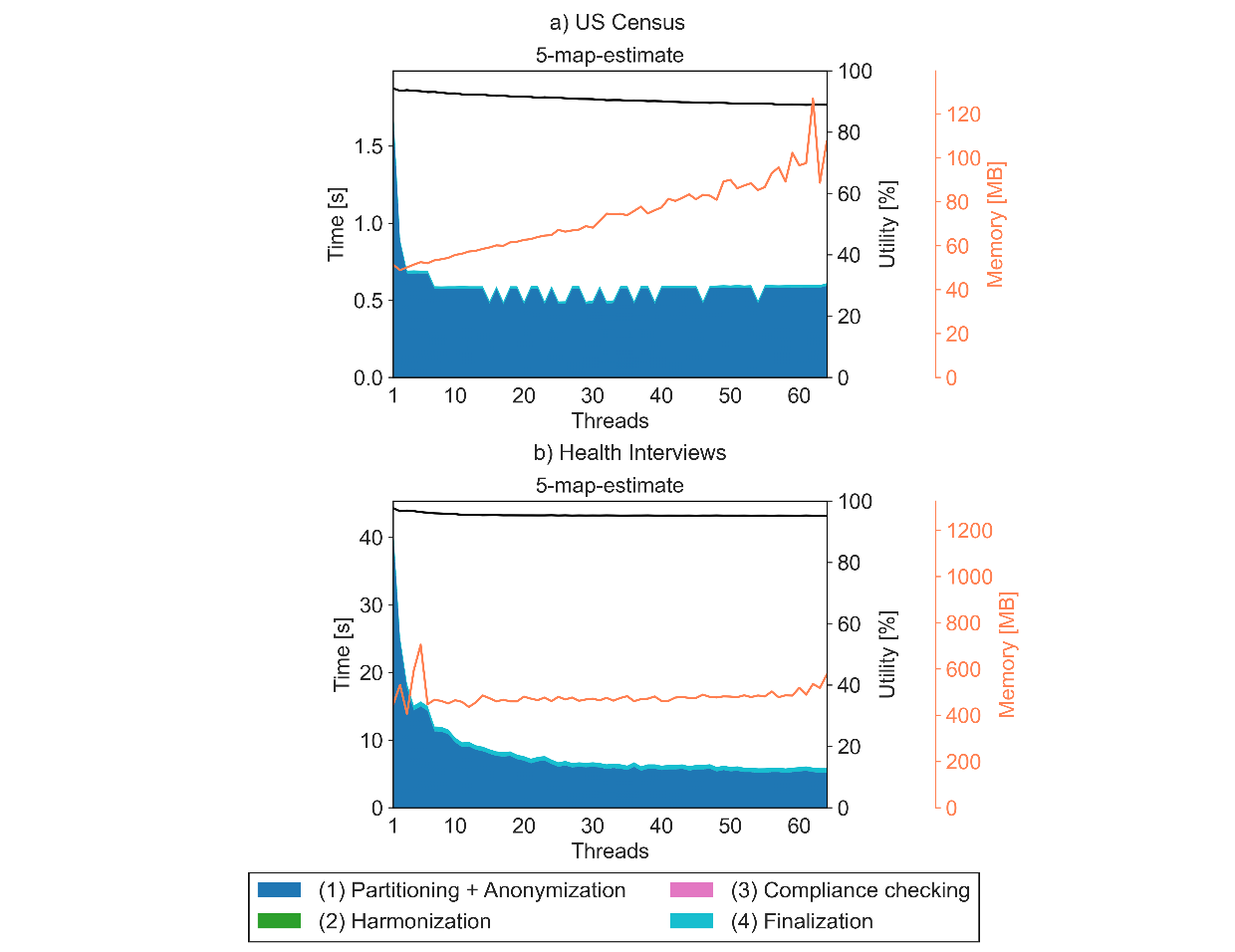

Figure S15:** Results for *k*-map runs with the transformation local setting. **
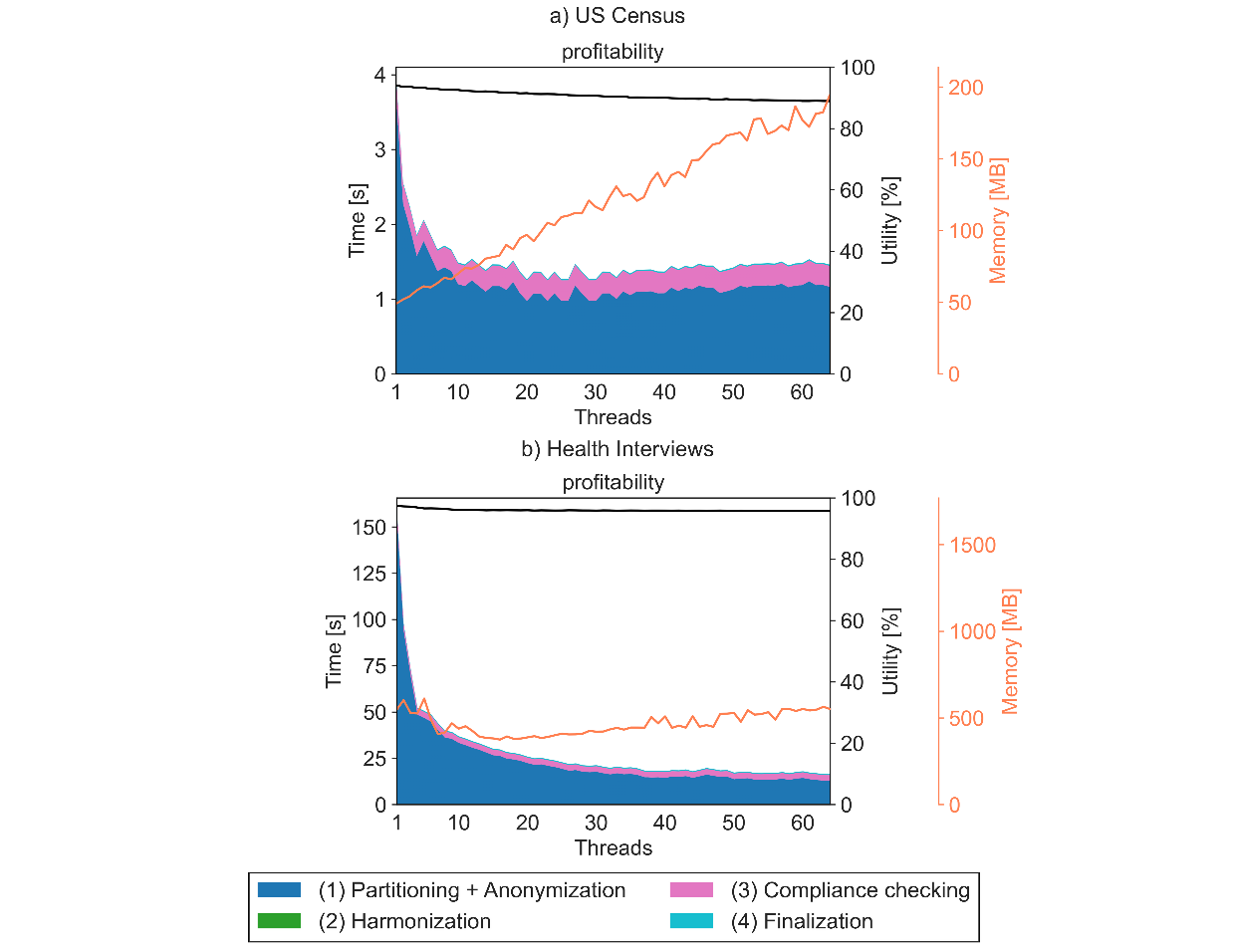

Figure S16:** Results for profitability runs with the local transformation setting.

# Dataset Configuration

In this section we describe the datasets used in our experiments and the anonymization settings for their individual attributes. The US Census dataset consists of 30,162 records with a total size of 2.46 MB, while the Health Interviews dataset contains 1,193,504 records and has a size of 93.06 MB. In the experiments, we used two different configurations. An overview is provided in Table S1 and Table S2.

| **Attribute name** | **Data type** | **Distinct values** | **Attribute type** | **Hierarchy levels** |
| --- | --- | --- | --- | --- |
| sex | Categorical | 2 | Quasi-Identifier | 2 |
| age | Numeric | 72 | Quasi-Identifier | 5 |
| race | Categorical | 5 | Quasi-Identifier | 2 |
| martial-status | Categorical | 7 | Quasi-Identifier | 3 |
| education | Categorical | 16 | Quasi-Identifier / Sensitive Attribute | 4 |
| native-country | Categorical | 41 | Quasi-Identifier | 3 |
| workclass | Categorical | 8 | Quasi-Identifier | 3 |
| occupation | Categorical | 14 | Quasi-Identifier | 3 |
| salary-class | Categorical | 2 | Quasi-Identifier | 2 |

**Table S1:** Overview of the nine attributes in the US Census dataset, including their data types, distinct values, attribute types and levels of the associated hierarchies.

| **Attribute name** | **Data type** | **Distinct values** | **Attribute type** | **Hierarchy levels** |
| --- | --- | --- | --- | --- |
| YEAR | Numeric | 13 | Quasi-Identifier | 6 |
| QUARTER | Categorical | 4 | Quasi-Identifier | 3 |
| REGION | Categorical | 4 | Quasi-Identifier | 3 |
| PRENUM | Numeric | 25 | Quasi-Identifier | 4 |
| AGE | Numeric | 86 | Quasi-Identifier | 5 |
| MARSTAT | Categorical | 10 | Quasi-Identifier | 3 |
| SEX | Categorical | 2 | Quasi-Identifier | 2 |
| RACEA | Categorical | 16 | Quasi-Identifier | 2 |
| EDUC | Categorical | 26 | Quasi-Identifier / Sensitive Attribute | 2 |

**Table S2:** Overview of the nine attributes in the Health Interviews dataset, including their data types, distinct values, attribute types and levels of the associated hierarchies.

As can be seen, both datasets consist of a mixture of categorical and numeric variables, with up to 86 distinct values. In the first setup, all attributes were considered to be quasi-identifiers. In the second setup, designed to study privacy models that protect sensitive attributes, one attribute was considered to be sensitive. The number of hierarchy levels describes the height of the domain-generalization hierarchy used. In ARX, hierarchies can be used to generalize values, suppress values or calculate distances as part of clustering processes [1]. When global transformation methods are being used, the product of all hierarchy heights determines the size of the search space that ARX must explore. Based on our configurations this size was 12,960 for the US Census dataset and 25,920 for the Health Interviews dataset.

# References

1. Prasser F, Eicher J, Spengler H, Bild R, Kuhn KA (2020) Flexible data anonymization using ARX—Current status and challenges ahead. Softw: Pract Exper 50:1277–1304

2. Xuyun Zhang, Yang LT, Chang Liu, Jinjun Chen (2014) A Scalable Two-Phase Top-Down Specialization Approach for Data Anonymization Using MapReduce on Cloud. IEEE Trans Parallel Distrib Syst 25:363–373
